# Participatory Patterns in an International Air Quality Monitoring Initiative

**DOI:** 10.1371/journal.pone.0136763

**Published:** 2015-08-27

**Authors:** Alina Sîrbu, Martin Becker, Saverio Caminiti, Bernard De Baets, Bart Elen, Louise Francis, Pietro Gravino, Andreas Hotho, Stefano Ingarra, Vittorio Loreto, Andrea Molino, Juergen Mueller, Jan Peters, Ferdinando Ricchiuti, Fabio Saracino, Vito D. P. Servedio, Gerd Stumme, Jan Theunis, Francesca Tria, Joris Van den Bossche

**Affiliations:** 1 Complex Networks and Systems Lagrange Laboratory, Institute for Scientific Interchange Foundation, Turin, Italy; 2 Department of Computer Science and Engineering, University of Bologna, Bologna, Italy; 3 Department for Artificial Intelligence and Applied Computer Science, University of Würzburg, Würzburg, Germany; 4 L3S Research Center, Gottfried Wilhelm Leibniz Universität Hannover, Hannover, Germany; 5 Institute for Complex Systems (ISC), CNR, Rome, Italy; 6 VITO—Flemish Institute for Technological Research, Mol, Belgium; 7 Extreme Citizen Science Research Group, Department of Civil, Environmental and Geomatic Engineering, University College London, London, United Kingdom; 8 Physics Department, University of Bologna, Bologna, Italy; 9 Physics Department, Sapienza University, Rome, Italy; 10 SONY-CSL Computer Science Lab, Paris, France; 11 CSP—Innovation in ICT, Torino, Italy; 12 Department of Electrical Engineering and Computer Science, University of Kassel, Kassel, Germany; 13 KERMIT, Dept. of Mathematical Modelling, Statistics and Bioinformatics, Faculty of Bioscience Engineering, Ghent University, Ghent, Belgium; Peking University, CHINA

## Abstract

The issue of sustainability is at the top of the political and societal agenda, being considered of extreme importance and urgency. Human individual action impacts the environment both locally (e.g., local air/water quality, noise disturbance) and globally (e.g., climate change, resource use). Urban environments represent a crucial example, with an increasing realization that the most effective way of producing a change is involving the citizens themselves in monitoring campaigns (a citizen science bottom-up approach). This is possible by developing novel technologies and IT infrastructures enabling large citizen participation. Here, in the wider framework of one of the first such projects, we show results from an international competition where citizens were involved in mobile air pollution monitoring using low cost sensing devices, combined with a web-based game to monitor perceived levels of pollution. Measures of shift in perceptions over the course of the campaign are provided, together with insights into participatory patterns emerging from this study. Interesting effects related to inertia and to direct involvement in measurement activities rather than indirect information exposure are also highlighted, indicating that direct involvement can enhance learning and environmental awareness. In the future, this could result in better adoption of policies towards decreasing pollution.

## Introduction

Air pollution has an important effect on our health, with an increasing number of studies showing higher risk of respiratory and cardiovascular diseases for people exposed to higher pollution levels [1, 2]. In this context, keeping air pollution at bay has been a major priority for policy makers in the past decades. A lot of effort has been put into monitoring and controlling air pollution. Large scale monitoring networks routinely monitor target pollutants. They allow for temporal trends in air pollution to be tracked. Significant effort has also been made to make information accessible to the wider public. However, several papers indicate that official monitoring networks do not have sufficient spatial coverage to provide detailed information on personal exposure of people, as for some pollutants, this may vary substantially among micro-environments [3, 4], i.e., in urban, traffic-prone areas spatial variability is very high [5–7]. Several pollution sources have been addressed with success. However, persistent problems remain in urban areas, where traffic and domestic heating are important sources [8]. Next to the technical solutions (e.g., electrical mobility), people’s personal perceptions, behavior and choices play a major role in addressing these issues and facilitating change in a bottom-up manner. This includes wide adoption of technologies developed for monitoring, which is mandatory in order to enable relevant results.

Participatory sensing, involving citizens in environmental monitoring, can have multiple potential benefits. Firstly, it can increase coverage of monitored areas, both in time and space, due to the ability to distribute the monitoring activities to multiple individuals [9]. Secondly, the act of monitoring pollution by citizens could facilitate learning and increase their awareness of environmental issues [10]. A recent report on environmental citizen science concludes that few studies on public participation in science and environmental education have rigorously assessed changes in attitudes towards science and the environment, and environmental behaviors. There appear to be relatively few examples of participatory citizen science having a tangible impact on decision making, although the potential is often noted [11].

One element to foster large scale participation in participatory monitoring campaigns is the availability of low-cost wearable sensing devices. These will give intrinsically lower quality data, since low cost implies decreased sensor accuracy, so at the moment the tradeoff is between the additional social benefits stemming from large participation and data quality [12]. As technology advances, low cost sensors will become increasingly more accurate, so that the tradeoff will disappear and the additional social effects will be obtained with no cost on data quality. Several efforts have been made to develop low-cost wearable sensing devices, integrating low-cost gas sensors, GPS and mobile phones. The CommonSense project [13] built hand-held devices containing CO, NOx and ozone sensors. Another example, which was quite successful in raising funds through crowdfunding, is the Air Quality Egg [14], designed for static measurements and containing NO_2_ and CO sensors.

However, many of these projects focus mainly on the electronics and systems integration, power issues, wireless data transfer, data storage and visualization and pay little attention to the limitations and quality issues of the gas sensors adopted. Very few tests or validation results have been published in publicly available reports or peer reviewed literature. Examples are Hasenfratz et al. and Mead et al.. Hasenfratz et al. [9] introduce GasMobile, a platform measuring ozone concentration, which is connected to a smartphone by USB. They take into account important issues such as sensor quality, calibration, and effect of mobility on sensor readings. Mead et al. [15] developed sensor boxes with electrochemical sensors, which entailed changes in the sensor technology itself, in the electronics and complex data analysis. The CitiSense [16] project is currently building an infrastructure for citizen engagement in environmental monitoring.

Another issue is the collection of a representative data set using mobile air quality sensing technologies. To be representative and useful for personal or community decision making, mobile measurements have to be repeated regularly, data have to be aggregated over relevant time frames and locations, and carefully interpreted using data handling and expert knowledge to filter out inaccuracies [6, 17]. The supplementary material S1 File discusses the challenges involved in using low-cost sensors for air quality monitoring and describes the approach used by our project to address quality issues.

An important issue concerns the technological versus social aspect of such projects. Most of the existing projects concentrate mainly on the sensor side of participatory air quality sensing, i.e., how to build the sensing devices and map pollution. However, participant engagement, participatory patterns, learning and awareness are equally important aspects, and feed back into the quality of the data collection, as we have also shown in a parallel project concerned with noise pollution [18]. By collecting subjective data as well, monitoring campaigns can enable not only air quality data collection, but also analysis of volunteer behavior, strategies and a possible increase in awareness.

### The test case

In this paper, we discuss the behavior and perceptions of citizens involved in monitoring, during a large scale international test case: the AirProbe International Challenge (APIC) [19]. This was organized simultaneously in four cities: Antwerp (Belgium), Kassel (Germany), London (UK) and Turin (Italy). In this test case a web-based game, air quality sensing devices and a competition-based incentive scheme were combined to collect both objective air quality data and data on perceived air quality, to analyze participation patterns and (changes in) perception and behavior of the participants. The test case was organized as a competition between the cities, to enhance participation. For the first time to our knowledge, an end-to-end scientific platform for participatory air pollution sensing, developed as part of the EveryAware project [20], was used. This platform is described briefly in the Methods section, with more details included in S1 File. The quality and representativeness of the collected air quality data are also discussed in S1 File.

During this test case, volunteer participants were asked to get involved in two activity types. The first one consisted in using a sensing device (Sensor Box), to measure air pollution (black carbon (BC) concentrations) in their daily life, generating what we call *objective* data. The second activity was playing a web game (AirProbe), where volunteers were asked to estimate the pollution level in their cities by placing flags (so called *AirPins*) on a map and tagging them with estimated black carbon (BC) concentrations on a scale from 0 to 10 *μ*g/m^3^, resulting in *subjective* data on air pollution (perception). Volunteers involved in the measuring activities were encouraged to play the game and bring other players as well (create a team).

The two data types allow for an analysis of user behavior and perception throughout the challenge. To enable this, the test case was composed of three phases. In phase 1, only the online game was available, so we could obtain an initial map of the perceived air pollution. In phase 2 the measurements started in a predefined area in each of the cities (corresponding also to the web game area), with the web game running in parallel. Phase 3 introduced a change in the game, so that players could acquire limited information about the real pollution in their cities in the form of sensor box measurements averaged over small areas (so called *AirSquares*). At the same time, measurements were continued, this time without a restriction of the area to be mapped. Incentives in the form of prizes were given at the end of each phase to the best teams/players (please see Methods and S1 File for more details).

The data collected during the test case are used here to analyze participation patterns, in terms of activity and coverage, and any changes in perception. Our results indicate that better coverage is obtained when volunteers are assigned a specific mapping area, compared to when they are asked to select the time and location of their measurements. Additionally, when allowed to measure freely, they seem to be attracted to places with higher pollution levels. Furthermore, while at the beginning of the challenge the general perception was that pollution was higher than in reality, perceptions changed in time indicating increased knowledge of real pollution levels. The amount of data collected in the test case, together with the first insights we obtained from it, suggest that bottom-up participatory sensing approaches are effective in attracting participants with high levels of activity and also in enhancing citizen awareness of real pollution levels.

## Results

Volunteer involvement and activity levels are among the most important elements in participatory monitoring campaigns, since these can determine the success of the campaign. Large activity is required for acquiring meaningful data, both objective, for analysis of the environment itself, and subjective, for analysis of social behavior. The test case presented here has successfully involved 39 teams of volunteers in 4 European locations, gathering 6,615,409 valid geo-localized data points during the challenge (the measuring device collects one data point per second). An additional 3,326,956 data points were uploaded to our servers in the same period, but were missing complete GPS information, and were not included in the analysis. Some of these measurements contained labels (tags), with 742 geo-localized overall tags coming mostly from one location of the challenge (London).

Additional information on perception of pollution has been extracted from the online game. The platform had 288 users in total, over six weeks, 97 of which played the game at least ten times. Their activity resulted in 70,758 AirPins at the end of the test case, which we will use to assess perceived pollution levels.

**Fig 1:**
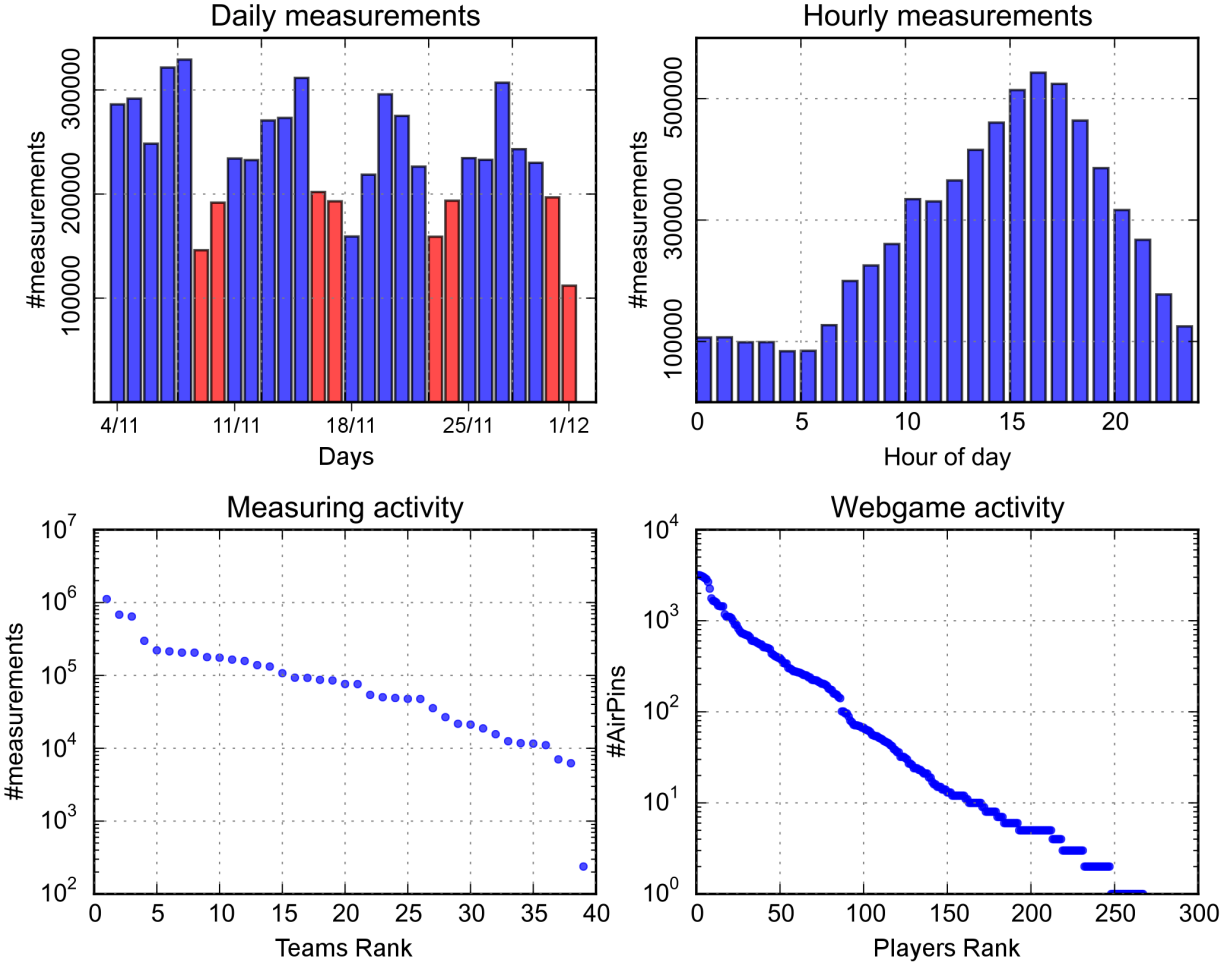
Volunteer activity patterns. The subplots in the top row show daily (weekends shown in red) and hourly measurements by volunteers. The distribution of the web game activity among players is shown in the bottom-right subplot, while the distribution of the number of measurements performed per team is depicted at the bottom-left (the distributions are displayed by ranking the volunteers by activity and then displaying the number of measurements/AirPins in descending order, using a rank-frequency plot).

Fig 1 shows general participation patterns, both for the measuring activity and for the web game. Further details about participation, for each of the four locations of the test case, can be found in S1 File. The daily number of measurements show larger activity during the week compared to weekends, with almost twice the activity in the peak days (Wednesday/Friday). This indicates that the volunteers were strongly interested in monitoring their exposure in relation to the routine activities of the week, which probably include commuting and access to highly polluted environments. It might also mean that it was easier for participants to monitor as part of their weekly routine whereby at the weekend monitoring would require more effort as it would not comprise part of their commute, for example, or may have impacted on other leisure activities that they wanted to carry out. Daily patterns (hourly measurements) indicate a peak in activity in the afternoon, around 5 pm, again probably due to afternoon commuting. However, measurements are performed at all hours of the day, indicating the presence of very dedicated volunteers. In fact, the total number of measurements per team indicates several teams with very high activity levels, with the most active team reaching almost 1 million points (equivalent to over 270 hours of measurements). However, team activity was very heterogeneous, with some teams collecting much less data than the others. This heterogeneity was found within the same city (e.g., the highly active teams are spread over three of the four cities), indicating that differences in activity were in general based on personal predisposition and not location. However, some of the heterogeneity between the cities can also be explained by the differences in instructions, emphasis and incentives.

The web game activity follows similar heterogeneous patterns. Fig 1 also shows the distribution of the number of AirPins used to declare perceived pollution levels by game players. Some of them got very involved in this activity, with over 2000 AirPins used, while many players had very low activity (started the game but did not continue). The distributions appear to follow a power law, also typical for other social activity patterns [21, 22]. It is important to mention that managing hundreds of AirPins required a large amount of time to be spent in the game, indicating the high involvement levels that the players reached.

Besides activity in terms of number of measurements, another important aspect is *coverage*, both in *space* and *time*. As we have seen before, measurements have been performed at all hours of the day and days of the week. However, usually not all areas are covered equally. Here we show general information about overall coverage achieved (with more details for each location included in S1 File).

**Fig 2:**
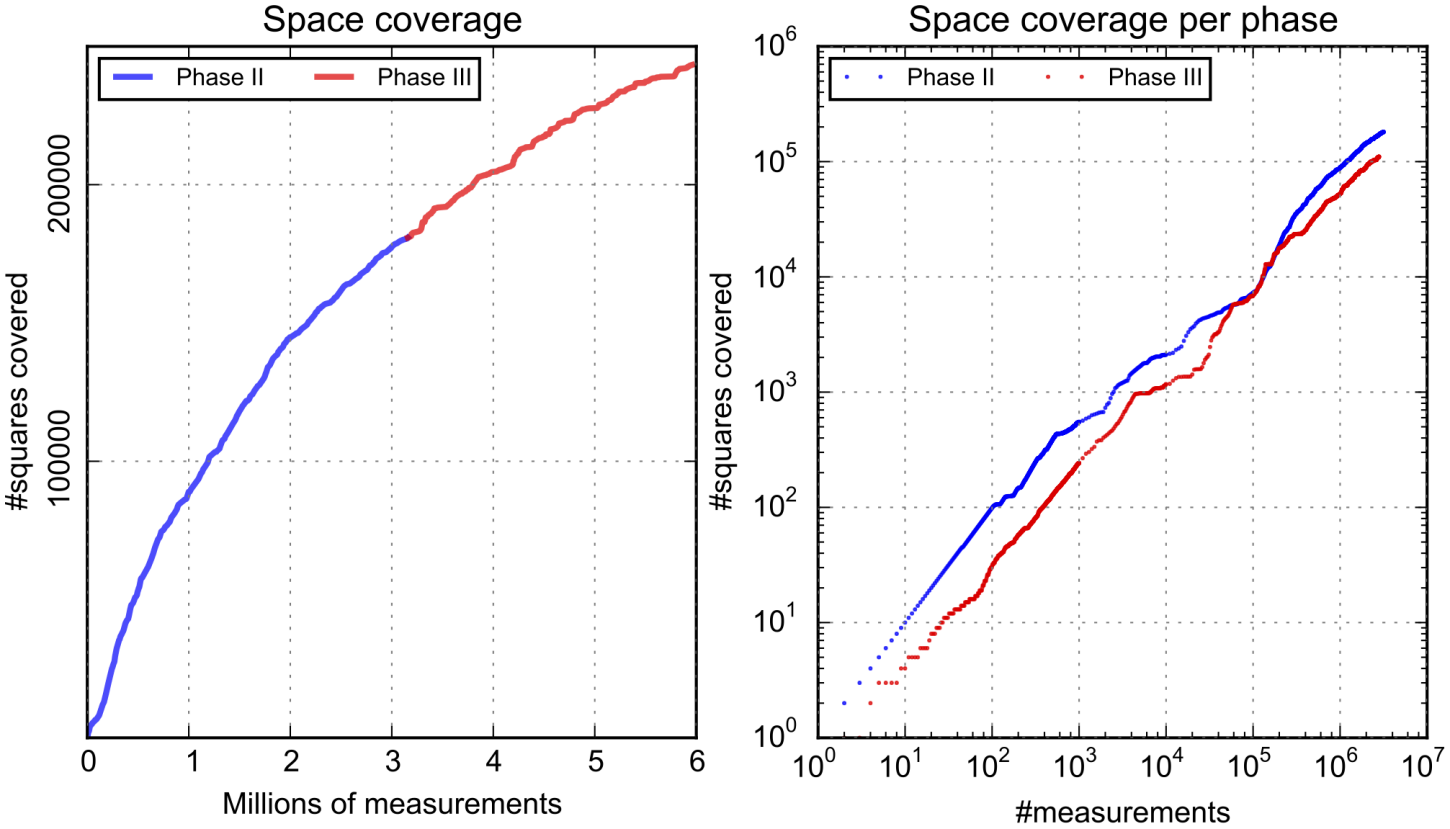
General space coverage data. Left panel: growth of the number of squares covered for the entire challenge. Right panel: growth of the number of squares covered per phase, in a log—log plot.

In order to compute the coverage, the area of each of the four participating cities was divided into 10 by 10 meter squares (tiles). Phase 2 mapping areas were selected to be around 2 km^2^, so the tiling resulted in about 20,000 such squares per location (80,000 in total). However, when computing coverage we selected larger areas that cover most of the surface of the 4 cities and encompass most measurements. Thus, the resulting number of squares considered was of 14,150,070. One square was considered covered if at least one measurement was performed within it. Fig 2 shows how the number of squares covered grows as users perform more measurements, both overall and for each phase individually. The volunteers had different tasks in the two measuring phases (phase 2 and 3 of the test case). In phase 2, they had to concentrate on covering as much as possible of a specific area, while in phase 3 they could explore any area they wanted. The total number of squares covered at the end of the challenge was over 243,000, i.e. over 24 km^2^, which is three times more than the mapping areas. If compared to the total surface of the cities considered, coverage is 1.7% only, but this depends a lot on the fact that some of the locations are very large, while the number of teams was comparable across the cities.

Fig 2 indicates that space coverage grows steadily with the number of measurements, meaning that users continue to explore new areas over the course of the challenge. However, while at the beginning of the challenge the growth is fast, this decreases in time. This indicates less exploration as the challenge evolves, due to the fact that volunteers measure at the same location multiple times. When looking at individual phases, it appears that during phase 2 space coverage was much better than in phase 3. This does indeed mean that volunteers displayed a better exploratory behavior at the beginning and when asked to cover a specific area of the city, compared to when they were asked to map any place they wished. In the latter case, they went for their daily routes that were not so extensive, and did not explore further. For both phases the growth of the space coverage follows a power-law, with exponent 0.73 in phase 2 and 0.79 in phase 3. This suggests that, although on the short term, space coverage in phase two is larger, in the long run the strategy of phase 3 might actually produce better coverage. However, the restricted time frame of our challenge can not provide further proof for this hypothesis.

**Fig 3:**
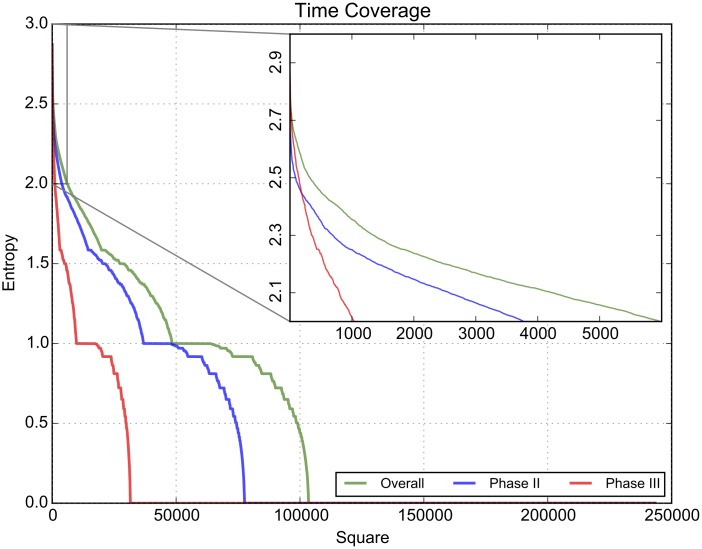
General time coverage data. Time coverage per phase and overall. The inset shows an enlarged view of the leftmost part of the plot (top ranked squares).

Since pollution levels vary both in time and space, it is important to have more measurements in the same location. So, for each tile, we also look at how measurements are spread in time, i.e., time coverage. We divided the measurements into 8 categories based on the time of measurement. First we separated the working days (Monday to Friday) from the weekends (Saturday and Sunday). Each of the two groups were divided into 4 further categories, by setting time thresholds at hours 08:00, 14:00, 18:00 and 23:00. The entropy of the resulting sets was computed. For each square, we obtained the fraction *f*_*i*_ of measurements in each category *i* as the ratio between measurements falling into that category and the overall number of measurements in that square. Then the entropy for that square is $S=-\sum_{i=1}^{8}\;f_{i}\;{\text{log}}_{2}\;f_{i}$. A higher entropy indicates a better spread of measurements in time. Fig 3 shows the distribution of the entropy for all squares covered, in a rank-entropy plot (squares are sorted descending by entropy and the entropy values plotted for each square). A few squares had a very good time coverage. These correspond to hubs in the four cities such as popular leisure locations (e.g. Königsstrasse in Kassel), main squares (e.g. Piazza Castello in Turin) and transportation hubs (e.g. the Barbican and Bank subway exits in London). At the other extreme there are many squares (more than half) that have been covered only in one time slot (entropy is 0). Between the two extremes, time coverage is dropping fast when moving through the ranked squares.

The curves display jumps and it appears that squares can be divided into sets based on time coverage. One first set (rightmost) includes those squares that have measurements only at one time of the day (entropy 0), which is followed by those covered in 2 time slots, ending with those that are covered at all times of the day (leftmost). Within each set, coverage decays differently. While for the highly covered squares decay appears to be exponential (as plotted in the inset), this becomes slower as the coverage decreases, with curves resembling polynomial decay.

When comparing the two phases, time coverage in phase 2 is much better overall than in phase 3. This indicates that volunteers not only explored more in space, but also in time, during phase 2, while in phase 3 they followed their daily schedule which allowed for poor time coverage as well. This underlines again the importance of giving volunteers a specific mapping area in order to obtain better measurement spread.

**Fig 4:**
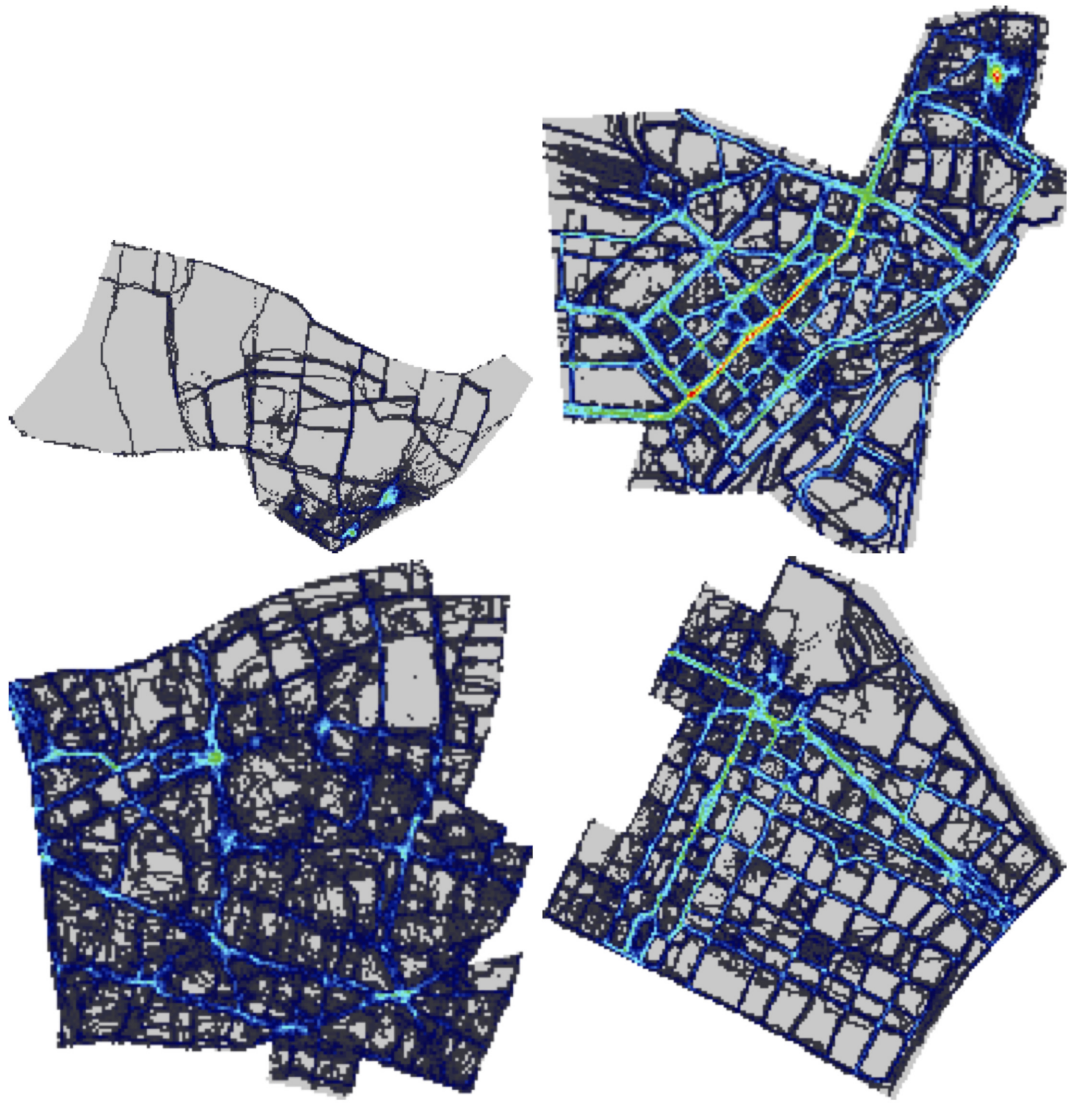
Coverage for phase two. Heatmap representation of time and space coverage in the 4 cities after the second phase of the challenge. Red dots represent strongly covered tiles. Only the mapping area for each city is represented. The cities are, from top left to bottom right: Antwerp, Kassel, London and Turin.

**Fig 5:**
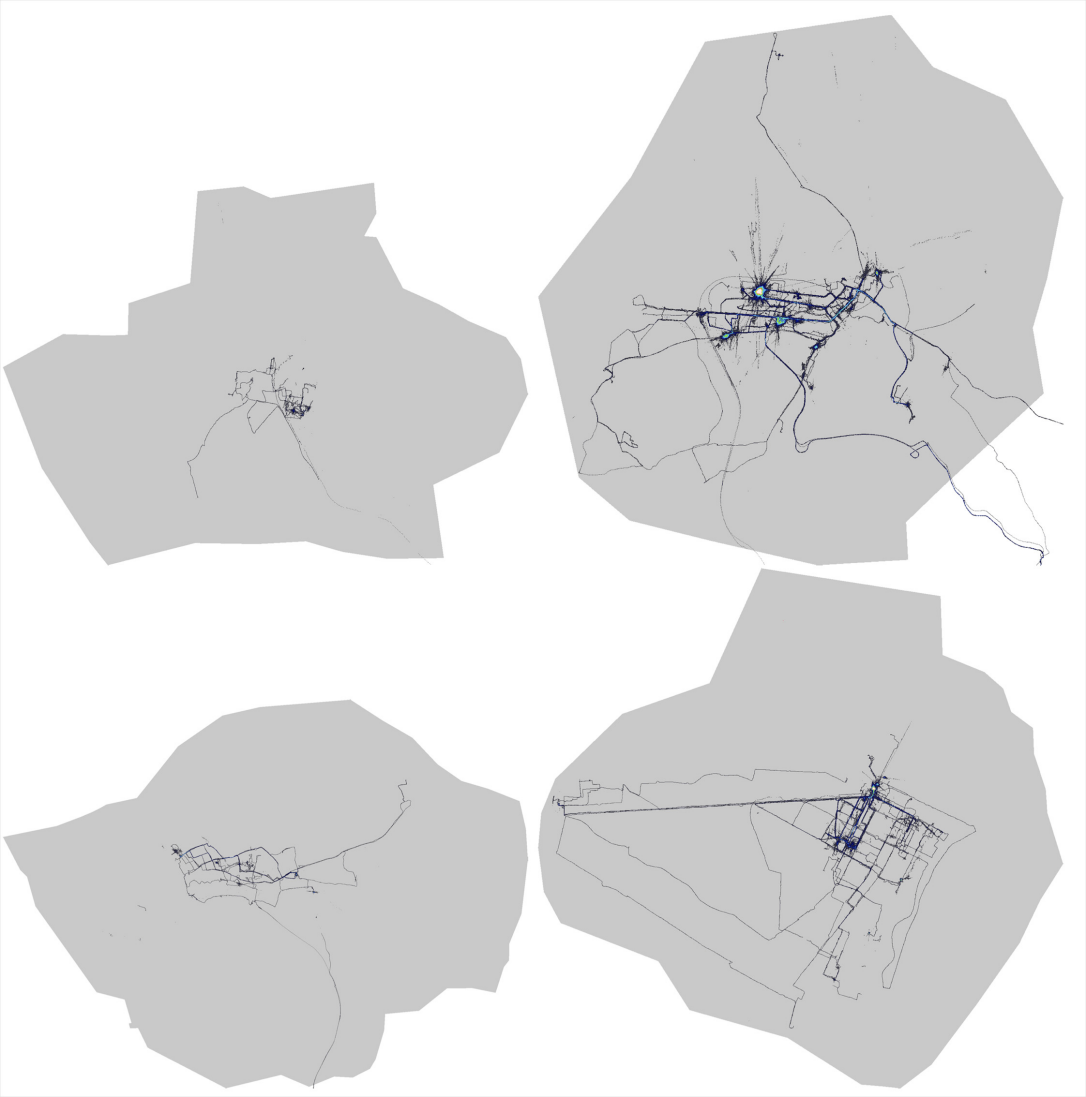
Coverage for phase three. Heatmap representation of time and space coverage in the 4 cities after the third phase of the challenge. Red dots represent strongly covered tiles. The entire area of the 4 cities is represented. The cities are, from top left to bottom right: Antwerp, Kassel, London and Turin.

The overall coverage results are also displayed as spatial heat maps in Fig 4 (phase 2) and Fig 5 (phase 3). These show the areas of the 4 cities (mapping area for phase 2 and the entire city for phase 3) with the covered tiles. Bright colours correspond to higher time coverage, with bright red indicating the locations with most measurements. It is clear that the mapping area (phase 2) is much better covered than others (phase 3), with a few clear locations containing many measurements. These do correspond to landmarks and main roads in the 4 cities, as discussed earlier.

**Fig 6:**
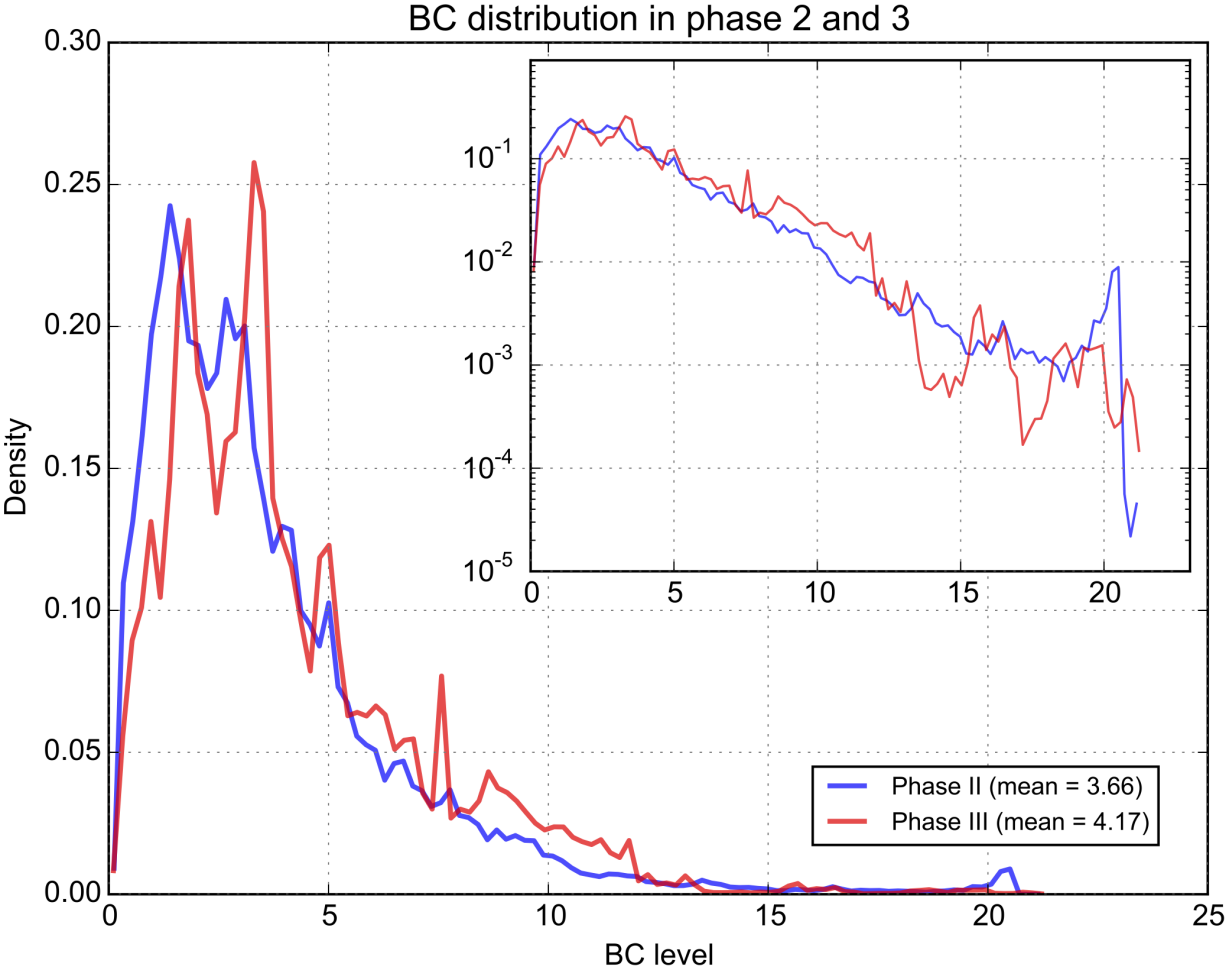
Overall pollution levels compared between the two phases. The distribution of BC levels are shown for the two measuring phases of the challenge. The vertical axis shows the density, i.e. the normalised frequency of the various BC levels measured, so that the area under the plot is 1. The inset shows the same plot but with a logarithmic vertical axis, to emphasize the tail of the distribution.

The measured BC levels can also provide useful insight into the aims and strategies of the volunteers during the challenge. To this end, we can examine how these change from phase 2 to phase 3. Thus, Fig 6 shows graphs of BC levels measured in the two phases, and we can observe larger BC values in phase 3 (the distribution is shifted to the right). A Kolmogorov-Smirnov test was performed to test whether differences are significant and a p-value of 2.2e-16 was obtained, confirming the difference. When volunteers can freely choose where to take measurements, it appears that they primarily target more polluted areas. When the mapping area is restricted, they tend to have a more systematic approach and cover lower pollution levels as well. One may argue that pollution levels may change naturally from one day to another, so the shift we see could be due to a higher average pollution level from phase 2 to phase 3. However, comparison with reference data seem to suggest that this is not the case (S1 File). Additional comparisons per location are also included in S1 File.

**Fig 7:**
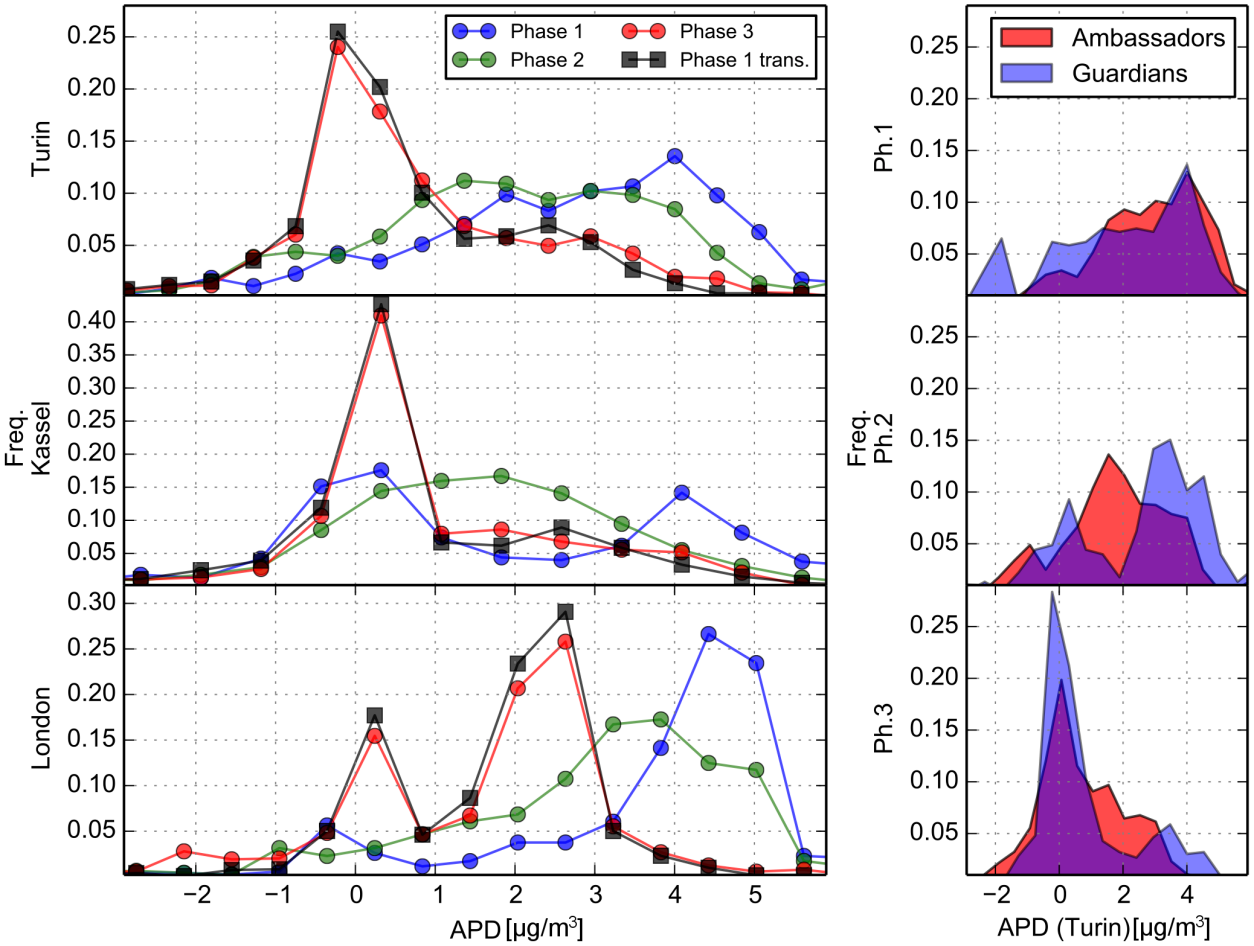
Web game data: APD distributions. APD is the deviation between the air quality level annotated (the AirPin value) and the aggregated measurements from sensor boxes (the AirSquare). The left part shows the distributions of the deviations in each phase for Turin, Kassel and London. An estimation of phase 3 distribution elaborated from phase 1 data with our model is also shown (Phase 1 trans.). The right part shows the distributions for Turin in each phase for AirAmbassadors (volunteers with sensor box that played the web game) and AirGuardians (only web game players).

The analysis of the structure and location of the collected objective data gives some insight into volunteer behavior and interests when measuring air pollution. Subjective data, on the other hand, can provide a stronger indication of changes in perception. For this, we look at the data collected by the web game, which consists of perceived levels of pollution in the mapping area, the AirPin values. In particular, to inspect awareness improvement and the learning process, we are interested in the relation between these annotations and the ‘true’ pollution values available in the web game during phase 3 in the form of AirSquares. Thus we define the APD (AirPin difference) as the difference between the AirPin value (perception of the volunteer) and the relative AirSquare value (real pollution level). In other words, the APD is the amount of ‘error’ in the annotation intended as distance from the measurement. Fig 7 shows several distributions of the APD. In the left part we have APD distributions in each phase for Turin, Kassel and London. Antwerp did not reach the critical mass of data required for this analysis (the number of web game volunteers was very restricted).

In phase 1, when no volunteer had been exposed to real measurements, we observe three different opinion structures in the three cities, representing the initial perception of volunteers. A systematic overestimation of pollution is present, i.e., the APD has peaks at ∼ 4 *μ*g/m^3^. This is likely to be caused by a scale misunderstanding: players, which were not accustomed to the BC concentration scale, almost ignored completely which values were to be considered reasonable and thus used the middle of the scale (i.e., 5 *μ*g/m^3^) as a ‘normal’ value. This results in the observed overestimation since the real average BC concentration measured lies between 1 and 2 *μ*g/m^3^.

In phase 2 things began to change. Some volunteers (so called *Air Ambassadors*) were given the sensor boxes to start performing measurements. The web game players consisted of these volunteers plus a set of other players recruited by them (so called *Air Guardians*). No data, except for the direct feedback from the boxes, was shown to the volunteers. Even so, a change is visible in the distribution of APD reported in the left part of Fig 7. By observing the measurements from their sensor boxes, Volunteers learn that in general BC concentrations are lower than what they believed, and respond by changing the values of the AirPins or taking the information into account when placing new ones. Since the change is quite significant, we also believe that those volunteers with the sensor boxes spread the information about what they were measuring, so that all players changed their perception. This decrease in the pollution levels reported in the subjective data of phase 2 is a first strong indication of learning during this phase. The right side of Fig 7 shows APD distributions separately for AirAmbassadors (performing measurements) and AirGuardians (who had no direct exposure to measurements until phase 3). We analyzed just the Turin dataset because in the other cities there was no clear distinction due to Ambassadors sharing their sensor boxes. The opinion shift in phase 2 is very strong for AirAmbassadors, but some change is also visible for AirGuardians, at least for part of the AirPins. This indicates that there was interaction among players, so that not only volunteers performing measurements, but some of their friends also, changed their perceptions.

Phase 3 brought an important change in the web game. AirSquares were made available, so players could acquire aggregated information (punctual information would have been just copied by the users) in form of average pollution levels within the respective square measured by the sensor boxes. There is a corresponding radical change in the subjective air pollution estimation emerging clearly in the left part of Fig 7. In all cities, there is a peak around zero in phase 3 in the APD distribution, meaning there were more players estimating the air quality correctly. This was in some way expected, since we are giving strong hints about pollution levels by means of AirSquares, but there is something more happening. In London there is another bigger peak and also in the other cities the distributions show some asymmetry, pointing out that people are not trusting the hints completely because in that case the distribution would have been more similar to a delta function, i.e., narrow and symmetric.

In order to describe this phenomenon we defined a stochastic transformation to reproduce the APD distribution for phase 3 starting from the APD distribution of phase 1. This transformation should reproduce the effect of the hints received by our volunteers on the initial distribution of their errors. Based on the empiric observation, the transformation takes into account two main effects: the possibility of complete trust in the hint, so that the opinion is reset near the hint, and the possibility of incomplete trust, so that the opinion is just shifted closer to the hint. The mathematical definition can be found in (S1 File). The left part of Fig 7 shows, for each location, how the transformed phase 1 data (black squares) matches phase 3 distributions, and this has also been confirmed with statistical procedures described in Methods and in S1 File. This provides an indirect proof of the assumptions of our model on the effect of objective data (complete and incomplete trust). Also, we were able to measure the ‘trust’ in the hints for the three cities, by fitting the model to data. We obtained the lowest trust values in London and the highest ones in Turin (full results are reported in S1 File).

## Discussion

Volunteer participation is crucial for the success of bottom-up monitoring campaigns, however most projects concerned with air pollution monitoring concentrate only on the development of the technical tools necessary. Here, we give a different user-centric perspective, using the experience from the EveryAware project, through its large scale international challenge, APIC. The tools developed by the project are described in more detail in S1 File. During the challenge both objective and subjective data were collected, and used here to analyze participatory patterns and possible changes in behavior or perception.

Objective measurements allowed for analysis of user interests during the challenge and activity patterns. A large number of measurements was obtained, however, coverage varied from location to location, with higher values when monitoring areas were restricted. Both coverage and pollution levels measured indicated a volunteer tendency to monitor familiar areas when there was no restriction, with a search for highly polluted spots.

Subjective data, on the other hand, allowed for analysis of perceived pollution levels and learning mechanisms. We observed, by analyzing differences between perceived and real pollution levels, that users are able to reduce the ‘errors’ in the annotations, by learning the true values. However, some inertia in changing the old opinion structure was also observed, since asymmetric tails and slow shifts of old peaks are present. We also looked at differences between AirAmbassadors (volunteers with sensor boxes that played the web game) and AirGuardians (only web game players). In phase 1 there is no clear distinction between them, as it is expected. In phase 2 Ambassadors, who begin to learn real pollution levels from the sensor boxes, start to shift their opinions, reducing the errors, while Guardians change less. Finally, in phase 3 we observe Ambassadors continuing to shift their opinions in a smooth way, with a certain inertia, while Guardians change radically showing a prominent primary peak at zero estimation error with a secondary peak in the position of the old peak. We can argue that the personal experience of the Ambassadors produces a smoother transition (which begins in phase 2), while the in-game information produces radical changes. But still both approaches shows the inertia we described earlier, even if in different forms.

In general, we can conclude that all our evidence shows that involving volunteers in monitoring campaigns can result in large amounts of data collected. These data show that participation can help learning, to create a more accurate perception of air quality. Thanks to our case study, it has also been possible to outline some of the mechanisms behind the resistance of subjective opinions to objective results.

Based on our experience, we can also propose a set of recommendations for future similar studies. First, the delineation of a mapping area is important, otherwise coverage is not uniform and becomes difficult to control. A second factor affecting uniformity of measurements is the length in time of the test cases. These should be at least a few weeks long, and ideally even spanning a few months, since at the beginning users tend to actively look for highly polluted spots. This is also important if behaviour shifts are expected, since just a few weeks are not enough to observe behaviour change. In terms of recruiting, our experience shows that upfront talks and events are most effective in attracting volunteers. While for enhancing awareness, we found that encouraging volunteers to recruit, among their friends, participants for some of the activities (the web game in our case) allowed for information from the sensors to spread also to volunteers not involved in the measuring activity. So, when the number of sensors is restricted, these other activities can facilitate the spread of awareness also outside the measuring group.

## Materials and Methods

The study presented here is based on data collected by volunteers during a large scale test case (AirProbe International Challenge—APIC) organized in four European cities (Antwerp, Kassel, London and Turin) in from October 2013 to November 2013. It required volunteers to measure air quality as well as provide their opinion on air pollution, using the EveryAware platform. This consists of a sensing device (Sensor Box), measuring air pollution, a mobile application (AirProbe), allowing for data visualization and upload to servers, a set of web services and websites, handling data storage and visualization and a web game developed on the XTribe platform [23], allowing to collect individual perceptions of pollution. In the following we provide a brief description of each of the components and of the tools used for data analysis, with further details included in S1 File.

### Ethics statement

This work is part of the European project Every Aware, contract number IST-265432. The European Commission finances only those projects that comply to its ethics and privacy regulations. Citing from the regulations of the Seventh Framework Programme, Decision No 1982/2006/EC, Article 6: “All the research activities carried out under the Seventh Framework Programme shall be carried out in compliance with fundamental ethical principles.” At the same time, the official rules for participation, Article 15, mention: “A proposal which contravenes fundamental ethical principles shall not be selected. Such a proposal may be excluded from the evaluation and selection procedures at any time”. Hence, acceptance and funding of this work by the European Commission implies approval of the ethics statement made in the proposal. This is why no further formal ethics approval was required for this research to be performed.

All participants to our study had to participate in training for using the sensor box and install our mobile application. Before admission to the test case, all volunteers were required to sign our Terms and conditions, which represents the user’s consent to use the measurements made. These clearly state that the data will be used for research purposes only and no personal information will be made public or used for other purposes. This includes sensitive information such as location data, names and contact information, that were collected during the test case.

Volunteers were recruited using a range of approaches in each city. These included a designated Facebook page, the EveryAware project website, posters, newspaper articles and either university mailing lists or those of local interests groups and environmental agencies (see the methods section ‘Case study’ and S1 File for further details). There was no specific inclusion/exclusion criteria used in the process. All volunteers could leave the study at any stage, however none chose to do so. There were 72 volunteers recruited in total, grouped into teams: 19 in Anwerp, 8 in Kassel, 35 in London and 10 in Turin. All volunteers named in the Acknowledgements section gave specific permission to be named.

### Sensing device: the sensor box

The sensor box contains a sensor array of 8 commercially available gas sensors and two meteorological sensors (temperature and humidity). The gas sensor array consists of low-cost continuous sensors of CO, NOx, O_3_ and VOC, which are important pollutants in the urban outdoor environments. These pollutants are either directly emitted by vehicles or other combustion processes, or formed from emitted precursors in the vehicle exhaust. The main criteria for sensor selection were the specific requirements posed by the mobile use of the sensor box for air quality monitoring as well as the hardware compatibility with the box. The gas sensors were examined by a range of performance tests under laboratory and outdoor conditions. These tests showed that none of the individual sensors can be used on its own. The observed selectivity, stability and response times of the different sensors introduced the need for a multivariate calibration procedure for the sensor boxes. Performance tests and calibration are described in more detail in S1 File.

**Fig 8:**
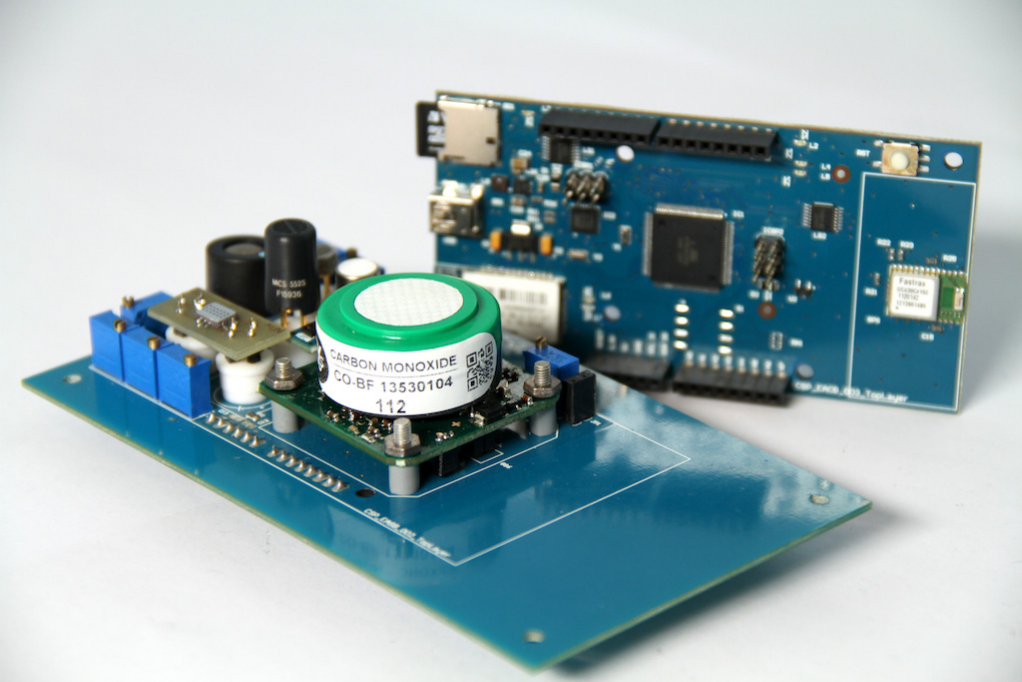
Sensing device. The two electronics boards of the sensor box with the gas sensors mounted on top of the sensor shield.

The sensor box electronic system has been designed with the purpose of being a low-cost, open and scalable platform. It is composed of two main boards (Fig 8). The first is a general purpose one that includes basic storage (micro SD card), positioning (GPS) and communication (Bluetooth) capabilities, while the second is a sensor shield able to host all gas sensors. The design is based on Arduino components and it is completely open source, so that anyone can reproduce and modify the hardware or even use the original hardware and develop different software to be run on it.

### The AirProbe mobile application

AirProbe is an Android application designed to connect to the sensor box via Bluetooth, acquire sensor readings and transit them to the EveryAware servers as soon as a working connection to the Internet becomes available. In addition, the application allows users to visualize the data they collect. Specifically, they can see their tracks on a map, calculate an estimated black carbon exposure and follow sensor output in real time plots. While collecting data, users can make free annotations (tags) that will be attached to the recordings and sent to the servers.

### Web platform

The case study web platform [24] is designed for collecting, storing, retrieving, analysing and visualizing large amounts of data data from different data sources. It provides endpoints for application like the AirProbe mobile application to upload data to. These data are then processed and cleaned, with several statistics and visualizations available on a public as well as a personal level. This facilitates further analysis and deeper understanding of the data by the user.

A collection of statistics pages provides overall information about the data, such as graphs showing currently active sensor boxes, the overall black carbon average per day, or the overall number of collected measurements per day. Also, information on separate sessions corresponding to different tracks (defined both by the Sensor Box and by the user) is available. This allows users to compare routes and locations. A world map gives a visual overview on the collected data. This includes cluster and grid views as well as a heatmap representation of the collected data on a personal as well as a global level providing visual information about areas with good measurement coverage and their average pollution levels. Users also have the possibility of downloading their own data, in case they want to compile any further personal statistics.

During the APIC challenge, the platform was specifically tuned for the needs of the game. Even though the platform supports several statistics and visualization of the data, most of this functionality has been disabled during the second stage of the challenge, in order to make opinions on air quality during the web game as unbiased as possible. The goal was for the AirAmbassadors and their sensor boxes to be the sole source of information regarding real measurements in order to limit information flow and facilitate a more controlled environment for the experiment. All visualisations were back online in the third phase of the challenge.

The web platform has been also providing a ranking page for the AirAmbassadors to be motivated throughout the challenge. Points were issued for space and time coverage during each collection phase. The ranking page showed which city and which team was ranked first globally as well as per city. In addition, the AirAmbassadors and their teams were able to access several statistics about their measurement behavior and the data collection process, including a coverage heatmap, the amount of covered squares and their points.

### The web game

The AirProbe web game is a simplified map management game. Players are called to fulfil their role of Air Guardians by annotating the map with so-called *AirPins*: geo-localized flags tagged with an estimated or perceived pollution level (black carbon concentration in *μ*g/m^3^, on a scale from 0 to 10). The game area of each city is divided into tiles. At the beginning of the game, users are asked to create a profile (by choosing an avatar and a name) and to choose a city and a team. Then the volunteer starts from a given tile of the map of the chosen city. Users can interact by placing (or editing or removing) AirPins or by expanding their territory, i.e., buying more tiles. Each day, the AirPins placed generate a revenue based on the precision of the annotation (precision depends on what other users think of the same area). In order to collect the revenue generated every day by each AirPin, the user has to access the game daily, otherwise the revenue will be lost. The collected revenue will be added to the user balance, allowing them to buy more AirPins and more tiles. In this way, players can build their air pollution perception map. At the beginning of phase 3, a new feature was made available in the web game: the AirSquare map. This consisted in an alternative map on which players could buy AirSquares, i.e., information about measured pollution levels aggregated on a small area. This data spreading stimulated the learning process described earlier.

### Case study

In order to set up the APIC study, volunteers were recruited in each of the four cities and they comprised two types of participants: Air Ambassadors, who were tasked with collecting air quality measurements with the sensor box, playing the online game, and recruiting Air Guardians, and Air Guardians, whose central focus was to play the online game and who were linked to a team of Air Ambassadors. Volunteers were recruited using a range of approaches in each city. These included a designated Facebook page, the EveryAware project website, posters, newspaper articles and either university mailing lists or those of local interests groups and environmental agencies (see S1 File for further details).

Incentives were offered during the initial call to participate in the study with the aim to encourage participation and maintain engagement. Prizes were given out to the team of Air Ambassadors with the best temporal/spatial air quality measurement coverage and the most active Air Guardians in each city over the different phases. Various strategies were incorporated into the online game to encourage ongoing play and the prizes related to the number of days played and the total revenue gained for each day of play. The rewards offered varied slightly across the four cities and are detailed in S1 File.

### Data analysis

To model the evolution between the phases of the APD distribution represented in the left part of Fig 7 (Phase 1 trans.), we implemented a simple modeling approach rearranging the opinions depending on their distances from the hint which is defined in S1 File. The transformation introduces 4 parameters, quantifying the inertia effects in the opinions shift. To check the quality of our model and to determine the values of parameters introduced we used a Kolmogorov-Smirnov test applied to the phase 3 dataset and to the phase 1 transformed dataset. Since it is a stochastic model, we performed several applications and found a convincing result for the *p*_*val*_ of 20%, which means that the hypothesis is consistent with observations. More details are provided in S1 File.

## Supporting Information

S1 File Platform description and further data analysis. Details for the different platform components and data features can be found in this file.
(PDF)

## References

[1] Raaschou-Nielsen O, Andersen Z, Beelen R, Samoli E, Stafoggia M, Weinmayr G, et al Air pollution and lung cancer incidence in 17 European cohorts: prospective analyses from the European Study of Cohorts for Air Pollution Effects (ESCAPE). Lancet Oncol. 2013;14:813–822. 10.1016/S1470-2045(13)70279-1 23849838

[2] Lave LB, Seskin EP. Air pollution and human health. vol. 6 Routledge; 2013. 10.1126/science.169.3947.723 5432570

[3] Dons E, Int Panis L, Van Poppel M, Theunis J, Wets G. Personal exposure to Black Carbon in transport microenvironments. Atmos Environ. 2012 8;55(0):392–398. 10.1016/j.atmosenv.2012.03.020

[4] Kaur S, Nieuwenhuijsen MJ, Colvile RN. Fine particulate matter and carbon monoxide exposure concentrations in urban street transport microenvironments. Atmos Environ. 2007;41(23):4781–4810. 10.1016/j.atmosenv.2007.02.002

[5] Peters J, Van Den Bossche J, Reggente M, Van Poppel M, De Baets B, Theunis J. Cyclist exposure to UFP and BC on urban routes in Antwerp, Belgium. Atmos Environ. 2014;92:31–43. 10.1016/j.atmosenv.2014.03.039

[6] Peters J, Theunis J, Van Poppel M, Berghmans P. Monitoring PM10 and ultrafine particles in urban environments using mobile measurements. Aerosol Air Quality Res. 2013;13:509–522.

[7] Setton E, Marshall JD, Brauer M, Lundquist KR, Hystad P, Keller P, et al The impact of daily mobility on exposure to traffic-related air pollution and health effect estimates. J Exp Sci Environ Epidemiol. 2011;21(1):42–48. 10.1038/jes.2010.14 20588325

[8] European Environment Agency. Air quality in Europe—2013 report; 2013.

[9] Hasenfratz D, Saukh O, Sturzenegger S, Thiele L. Participatory air pollution monitoring using smartphones. Mob Sens. 2012;.

[10] Assembly UG. Rio declaration on environment and development. Agenda. 1992;21.

[11] Science Communication Unit—University of the West of England—Bristol. Science for Environment Policy In-Depth report: Environmental Citizen Science; 2013.

[12] Cuff D, Hansen M, Kang J. Urban sensing: out of the woods. Commun ACM. 2008;51(3):24–33. 10.1145/1325555.1325562

[13] Dutta P, Aoki PM, Kumar N, Mainwaring A, Myers C, Willett W, et al. Common Sense: Participatory Urban Sensing Using a Network of Handheld Air Quality Monitors. In: Proceedings of the 7th ACM Conference on Embedded Networked Sensor Systems. SenSys’09. New York, NY, USA: ACM; 2009. p. 349–350.

[14] Tangient LLC. Air Quality Egg project, airqualityegg.com; 2013 Date of access: 01/02/2014.

[15] Mead MI, Popoola OAM, Stewart GB, Landshoff P, Calleja M, Hayes M, et al The use of electrochemical sensors for monitoring urban air quality in low-cost, high-density networks. Atmos Environ. 2013 5;70(0):186–203. 10.1016/j.atmosenv.2012.11.060

[16] Castell N, Kobernus M, Liu H, Schneider P, Lahoz W, Berre AJ, et al. Mobile technologies and services for environmental monitoring: The Citi Sense MOB approach. Urban Climate. 2014;.

[17] Van den Bossche J, Peters J, Verwaeren J, Botteldooren D, Theunis J, De Baets B. Mobile monitoring for mapping spatial variation in urban air quality: development and validation of a methodology based on an extensive dataset. Atmos Environ. 2015 Jan;.

[18] Becker M, Caminiti S, Fiorella D, Francis L, Gravino P, Haklay MM, et al Awareness and Learning in Participatory Noise Sensing. PLoS ONE. 2013 12;8(12):e81638 10.1371/journal.pone.0081638 24349102 PMC3859489

[19] Everyaware Consortium. AirProbe International Challenge - www.everyaware.eu/category/apic/; 2013 Date of access: 03/02/2015.

[20] EveryAware Consortium. EveryAware Project, www.everyaware.eu; 2012 Date of access: 03/02/2015.

[21] Garas A, Garcia D, Skowron M, Schweitzer F. Emotional persistence in online chatting communities. Nat Sci Rep. 2012;402:1–34. 10.1038/srep00402 PMC3349267 22577512

[22] Tria F, Loreto V, Servedio VDP, Strogatz SH. The dynamics of correlated novelties. Nat Sci Rep. 2014;4. 10.1038/srep05890 PMC5376195 25080941

[23] Caminiti S, Cicali C, Gravino P, Loreto V, Servedio VDP, Sirbu A, et al. XTribe: A Web-Based Social Computation Platform. In: Cloud and Green Computing (CGC), 2013 Third International Conference on; 2013. p. 397–403.

[24] Becker M, Mueller J, Hotho A, Stumme G. A Generic Platform for Ubiquitous and Subjective Data. In: 2013 ACM International Joint Conference on Pervasive and Ubiquitous Computing, UbiComp 2013; 1st International Workshop on Pervasive Urban Crowdsensing Architecture and Applications, PUCAA 2013, Zurich, Switzerland—September 8–12, 2013. Proceedings. New York, NY, USA: ACM; 2013. p. 1175–1182.

