# Supplementary material for: Participatory Patterns in an International Air Quality Monitoring Initiative
Source: PLoS One. 2015 Aug 27;10(8):e0136763. doi: 10.1371/journal.pone.0136763 (PMC4551740; doi:10.1371/journal.pone.0136763)
Supplement: S1 File — Details for the different platform components and data features can be found in this file. (PDF) [file pone.0136763.s001.pdf]

Participatory patterns in an international air quality monitoring initiative
Supplementary material

Alina Sîrbu, Martin Becker, Saverio Caminiti, Bernard De Baets, Bart Elen, Louise Francis, Pietro Gravino, Andreas Hotho, Stefano Ingarra, Vittorio Loreto, Andrea Molino, Juergen Mueller, Jan Peters, Ferdinando Ricchiuti, Fabio Saracino, Vito D. P. Servedio, Gerd Stumme, Jan Theunis, Francesca Tria, Joris Van Den Bossche

1 The EveryAware sensor box

All the air quality sensors (Table 1) were subjected to laboratory and outdoor tests for further characterization. In laboratory tests, sensors were exposed to synthetic gas mixtures (CO and NO_2) at a constant temperature of 25°C and a relative humidity of 50%. The sensors included in this analysis were the Alphasense CO-BF sensor, the e2v MiCS-5521 CO sensor and the e2v MiCS-2710 NO_2 sensor. The experiments were run for approximately 3 hours. The CO sensors were exposed to a series of CO gas concentrations of 9.18, 6.89, 4.61, 2.3 and 1.15 ppm. Between the different concentrations, a zero measurement was made. Each step of the measurement series lasted for approximately 20 minutes. The NO_2 measurements were made with concentrations of 85, 44, 24 and 0 ppb. The response times of the sensors (T90), defined as the time required for the sensor to reach 90% of its maximal value in response to a step change from zero to a certain concentration value, was monitored at a 30 second resolution. Average T90 response times were 150 seconds, 180 seconds and 270 seconds for the Alphasense CO-BF sensor, the e2v MiCS-5521 CO sensor and the e2v MiCS-2710 NO_2 sensor, respectively. The linearity of the sensors was high for the Alphasense CO-BF sensor in the 0 - 10 ppm CO range ($R^2 > 0.99$), and for the e2v MiCS-2710 NO_2 sensor for NO_2 concentrations between 0 and 90 ppb (R^2=0.98). The e2v MiCS-5521 CO sensor showed a non-linear relationship in the 0-10 ppm CO concentration range.

Because the controled laboratory setting is very different from outdoor conditions, the main tests were performed outdoor. The outdoor performance tests were carried out at a station from the Flemish air quality monitoring network. The station (Borgerhout, 42R801, see www.ircel.be) is situated at a traffic location along a double lane main street with an average daily traffic volume of 43,381 vehicles (42,961 cars and 420 heavy duty vehicles, data from the Traffic Centre Flanders). We used 4 sensor boxes for the outdoor performance tests from October 2012 until April 2013. By placing the sensor boxes at an official monitoring station we gained the advantage of having reference data for several pollutants (CO, NO, NO_2, O_3 and Black Carbon (BC)) albeit at a coarser temporal resolution of 30 minutes. The average gas (CO, NO, NO_2 and O_3) concentration and BC concentration during the outdoor tests are given in Table 2. A cross-correlation analysis was performed to compare the 30 minute averaged sensor data with the reference data for several pollutants (Table 3). Correlation between the reference data is given in Table 4 for comparison.

Low to moderate correlations were found between the CO sensor measurements and the

Table 1: Overview of the sensors of the sensor box.

Sensor	Measured parameter	Dynamic range	Cost
Alphasense CO-BF	CO		180 Euro
e2v MiCS-5521	CO	1 - 1000 ppm	3.4 Euro
e2v MiCS-5525	CO	1 - 1000 ppm	5 Euro
Figaro TGS 2201 (dual)	CO	10 - 1000 ppm	15 Euro
Figaro TGS 2201 (dual)	NOx	0.1 - 10 ppm	15 Euro
e2v MiCS-2710	NO_2	0.05 - 5 ppm	3.7 Euro
e2v MiCS-2610	O_3	10 - 1000 ppb	3.7 Euro
Applied Sensors AS-MLV	VOC	not available	5 Euro
Sensirion SHT21	temp	-40 - 125 °	15 Euro
Sensirion SHT21	rel. humidity	0 - 100 %	15 Euro

Table 2: Average concentration and standard deviation of CO, NO, NO_2, O_3 and BC by the reference monitors during the outdoor testing period.

	mean	stdev.
CO	0.32 ppm	0.13 ppm
NO	32.27 ppb	35.34 ppb
NO2	26.56 ppb	11.50 ppb
O3	9.87 ppb	10.00 ppb
BC	4.03 μg/m^3	2.73 μg/m^3

Table 3: Cross-correlation between sensor measurements and reference gas measurements or meteorological data measured inside the sensor box. Averages of 4 sensor boxes are shown together with the standard deviations between brackets.

	Reference monitors					sensor box	
Sensors	CO*	NO*	NO_2 *	O_3 **	BC*	temp (°C)*	% RH*
Alphasense CO-BF	0.52 (0.16)	0.41 (0.11)	0.34 (0.11)	-0.32 (0.14)	0.35 (0.13)	-0.81 (0.11)	0.00 (0.16)
e2v MiCS-5521 CO	0.31 (0.04)	0.32 (0.04)	0.34 (0.04)	-0.09 (0.11)	0.41 (0.02)	0.89 (0.06)	-0.214 (0.06)
e2v MiCS-5525 CO	0.60 (0.02)	0.51 (0.05)	0.56 (0.05)	-0.71 (0.05)	0.55 (0.06)	0.50 (0.06)	0.25 (0.03)
Figaro TGS 2201 CO	0.25 (0.02)	0.32 (0.01)	0.17 (0.00)	-0.48 (0.01)	0.38 (0.01)	0.45 (0.03)	0.46 (0.07)
Figaro TGS 2201 NOx	-0.78 (0.01)	-0.40 (0.06)	-0.24 (0.05)	0.47 (0.05)	-0.47 (0.06)	-0.40 (0.04)	-0.21 (0.03)
e2v MiCS-2710 NO_2	-0.58 (0.02)	-0.40 (0.06)	-0.31 (0.08)	0.64 (0.07)	-0.49 (0.06)	-0.40 (0.07)	-0.27 (0.02)
e2v MiCS-2610 O_3	-0.67 (0.06)	-0.56 (0.02)	-0.55 (0.05)	0.83 (0.07)	-0.62 (0.03)	-0.18 (0.15)	-0.12 (0.19)
Applied Sensors AS-MLV VOC	0.63 (0.02)	0.43 (0.17)	0.53 (0.15)	-0.44 (0.26)	0.45 (0.19)	0.23 (0.22)	0.14 (0.10)

* streetside
** backyard (30 m from street)

Table 4: Cross-correlation between reference gas measurements and meteorological data measured inside the sensor box (average of 4 sensor boxes).

	Reference monitors					sensor box	
	CO	NO	NO_2	O_3	BC	temp (°C)	% RH
CO	1.00	0.77	0.62	-0.55	0.83	-0.07	-0.09
NO	0.77	1.00	0.76	-0.51	0.89	0.13	-0.06
NO_2	0.62	0.76	1.00	-0.53	0.81	0.11	-0.24
O_3	-0.55	-0.51	-0.53	1.00	-0.54	-0.12	-0.22
BC	0.83	0.89	0.81	-0.54	1.00	0.23	-0.08

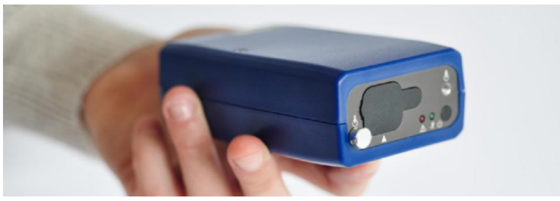

Figure 1: Micro-aethalometer: device used as a reference for calibration.

CO reference data. The Alphasense CO-BF and e2v MiCS-5525 CO sensors had the highest correlations (0.52 and 0.60, respectively) of the four CO sensors, although the Alphasense CO-BF sensor showed significant variability between sensors. The non-CO sensors showed higher correlations with the reference CO measurements. By sharing the same sources in the urban environment, it is logical that monitoring signals of different pollutants show high correlations (e.g. Table 4). The high correlations between the non-CO sensors and the reference CO data can therefore be explained. The Figaro TGS 2201 NOx and e2v MiCS-2710 NO_2 sensors showed a moderate correlation with NO and NO_2. The negative sign is due to the electronics and can be discarded in this analysis. Correlations of the NOx sensors are higher with CO and O_3, although these values stayed within the correlation range that was observed for the reference measurements. It is not proven by this experiment that the high correlation with CO and O_3 is due to selectivity problems of the sensor. The e2v MiCS-2610 O_3 sensor showed a high correlation with the reference O_3 measurements. Also the variability between the sensor boxes was limited. Correlations with other gases are negative, which is in line with the physical reality (see reference measurements in Table 4). The Applied Sensors AS-MLV VOC sensor shows the highest correlations with CO and NO_2, but reference VOC measurements are lacking. The correlation with BC ranges between 0.35 and -0.62. These correlations are in the same moderate range as the correlations that are found between the sensors and the reference measurements of the respective gases.

Important conclusions of these laboratory and field experiments with respect to further developments and applications of the sensor box are: (i) response times of the sensors are in the minute range rather than in the second range; (ii) correlations between sensor and reference measurements are low to moderate for most of the sensors, for the e2v MiCS-2610 O_3 sensor the correlation is high; (iii) moderate correlations between the sensor measurements and the BC measurements are observed.

2 Sensor box calibration

Following the analysis of sensor abilities presented in the previous section, we have proceeded with calibration of the sensor boxes. Issues identified by our initial analysis included sensor sensitivity to temperature and humidity, sensor drift in time and sensitivity to other gasses. Hence, one needs to calibrate devices against a reference in order to control for this issues and obtain a measurement meaningful for the user.

Supervised learning was employed to model an unknown concentration of a target pollutant from sensor array measurements (based on low-cost gas sensors, temperature and relative humidity sensors). The supervised learning model is parameterised by using a training dataset consisting of sensorbox measurements and simultaneous target pollutant concentration measurements. The target pollutant selected in this study was black carbon (BC). The selection of BC as a target was motivated by following three reasons: (1) BC is a relevant pollutant in urban

Table 5: Performance of the ANN, SVM and RF techniques to model BC from sensor box data on independent mobile data.

	R^2	rmse
ANN	0.26	2.10
SVM	0.23	1.70
RF	-0.13	1.72

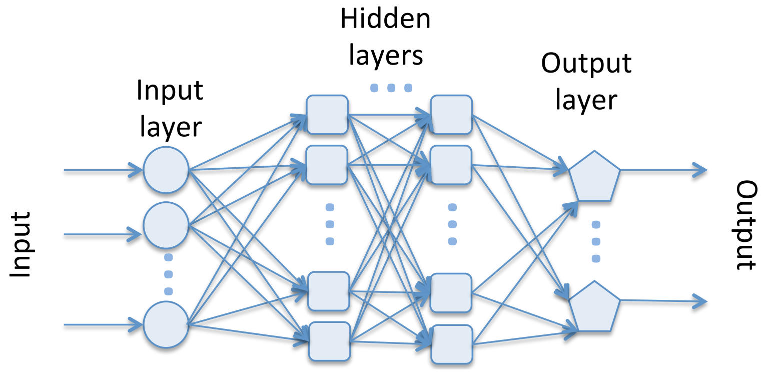

Figure 2: General structure of artificial neural networks.

environment by its adverse health effects [?], (2) BC is correlated with the gases that are measured by the sensor box, as seen in the previous sections, and (3) the availability of portable BC measurement devices (micro-aethalometers, AethLabs, Figure 1) which makes it possible to collect mobile BC data.

2.1 Calibration model

The first calibration datasets, consisting of sensor box data measured at the same time with micro-aethalometer data, were obtained in Antwerp near an air quality monitoring station at a traffic site and in Turin in spring 2013 from a two-week long monitoring with sensor boxes and micro-aethalometers positioned near a busy road. These datasets were used to compare the performance of different supervised learning techniques. We have explored four different possible models to use for mapping of sensor output to the reference measurements. These were Random Forests, Support Vector Machines (SVMs), a custom air quality index and Artificial Neural Networks (ANNs). After comparing these (Table 5), SVMs and ANNs obtained a similar behaviour, better than the other two options, but training of ANNs appeared to be faster, so we decided to adopt them for our model.

ANNs[?] are regression models that mimic the behaviour or neuronal networks. They consist of interconnected computing units (neurons) that can have several inputs and an output. In each unit, two operations happen: compute a weighted sum of the inputs and apply a sigmoid (activation) function to obtain the output. The network can have several layers feeding into one another: one input and one output layer plus a number of hidden layers (see Figure 2).

In order to train a network, one needs to select a topology and find the values of the input weights for each neuron. To select the topology we have performed an empirical analysis that led to the usage of a network with one hidden layer of 10 neurons. The final topology is displayed in Figure 3. After this, we used backpropagation, a standard algorithm for ANN training [?], to

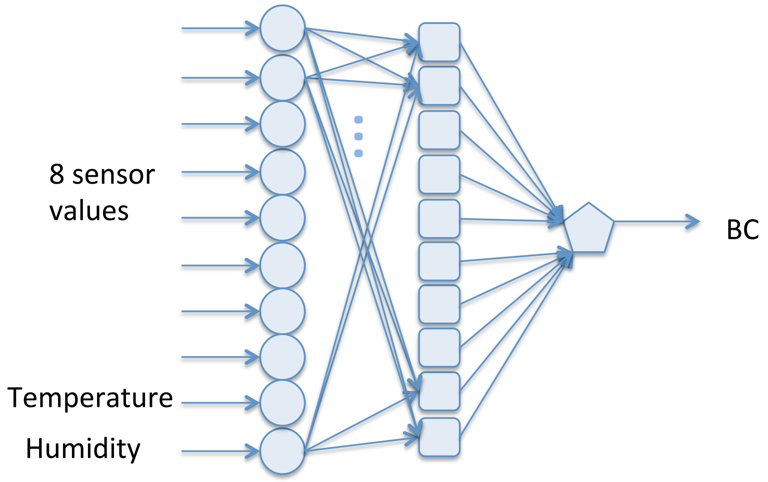

Figure 3: ANN topology for our calibration problem.

obtain the network weights.

The ANN model was implemented both in the AirProbe mobile application as well on the server. This was done to give the user real time feedback from the sensor box on the one hand, but on the other hand allow the mobile device to leave the computationally expensive calculation of the black carbon value to the server in case the user is not using the viewing of black carbon values in real-time.

This approach was taken due to the fact that the sensor box has two working modes, online and offline. Computing model output for all offline records would have been too computationally expensive for an average smartphone, while server side this was not an issue.

2.2 Preprocessing

Although initially the possibility of building one calibration model for each box was intended, this would not have scaled very well, so we explored the possibility of building one model for all sensor boxes. From the first calibration data sets we observed that sensor boxes behaved similarly when exposed simultaneously. Although the absolute sensor values differ between boxes, the fluctuations in time are similar for the same sensors. This means that sensor box rescaling (i.e. normalisation of the sensor signals) could be used to scale the different sensor boxes within the same range, and parameterise a model on the standardised data. This would mean obtaining a unique model for all sensor boxes, instead of individual ones for each box. For APIC we decided to use one calibration model for each city to account for possible differences in sensor response between locations.

A different issue was data variability, both in BC values and sensor response. The BC values were first processed by a noise reduction algorithm [?] to lower the high-frequency instrument noise that is observed when measuring at high frequency. To remove sensor box fluctuations, the sensor data was smoothed by computing averages over a moving time window of one minute. The resulting BC values were averaged over a 5 minute window. This value was deemed suitable by comparing outputs from two aethalometers, which become highly correlated at this resolution. So the BC value obtained from the model represents an average over the last five minutes of exposure.

2.3 Model performance

For each location of the challenge, models were trained on experimental data. Three types of data were used, to account for three possible use cases. These included stationary data where all sensor boxes were collocated, mobile measurements performed with one or two boxes at a time, and indoor data. Training and testing datasets were obtained by combining all these data types, and four models were obtained, one for each location.

Figures 4, 5, 6 and 7 display the best model obtained in each city, in terms of performance on training and test data, as well as cumulative exposure on the test data. The Turin model (Figure 7) performs best, and this is due to the increased amount of data, especially mobile, available for this location, since preliminary calibration tests were performed there. This demonstrates that the collection of large amounts of mobile data is crucial for boosting modelling abilities. The Antwerp dataset also contained larger amounts of mobile data, compared to the other two, so that a good performance was obtained as well (Figure 4). Although datasets were more restricted for Kassel and London, indications were that models obtained were displaying good performance (see Figures 5 and 6).

In general, calibration was successful at identifying general trends in the pollution levels. However, sharp and short peaks are not handled well by the model, and this is due to the lower sensitivity of the low-cost sensors and their delayed response. However, the performance obtained was enough for the purposes of our project, i.e. participatory mapping of pollution with multiple devices, for enhancing environmental awareness.

3 The AirProbe application

The AirProbe application is freely available for the Android platform and can be installed from Google PlayStore. The main objective of the application is to acquire the data from the sensor box and to upload it to the EveryAware server. The application also allows the user to view/annotate data and can operate in three different modes: Live Track, Synchronization and Browsing. Without this application, the sensor box data cannot be accessed nor uploaded to the servers.

In order to associate the data uploaded with a specific user, the application must be activated. This process links the application to an existing EveryAware account (which can be created inside the application itself or on the project web site).

3.1 Live Track mode

This is the standard way to use AirProbe to collect air quality measurements. In this mode, the application will search for Bluetooth devices nearby and present the user with a list of found devices. EveryAware sensor boxes can be easily identified by their MAC suffix. Once the user has selected the sensor box, AirProbe starts displaying real time data collected by the sensor box, using the Bluetooth connection. In Live Track mode, the interface is composed of three different views accessible from their corresponding tabs (Figure 8):

Map , where users can follow their own live track. The track is represented with different colours, depending on real-time black carbon levels. The user can also add annotations and share them on social networks (Facebook/Twitter), using the buttons at the top right corner. The track length to be shown on the map can be of 5, 15, 60 minutes. Live tracking of the current position can be switched on/off, through the top left buttons. The bar at the top represents the black carbon value using a coloured scale (from a blue/low value to a brown/high value).

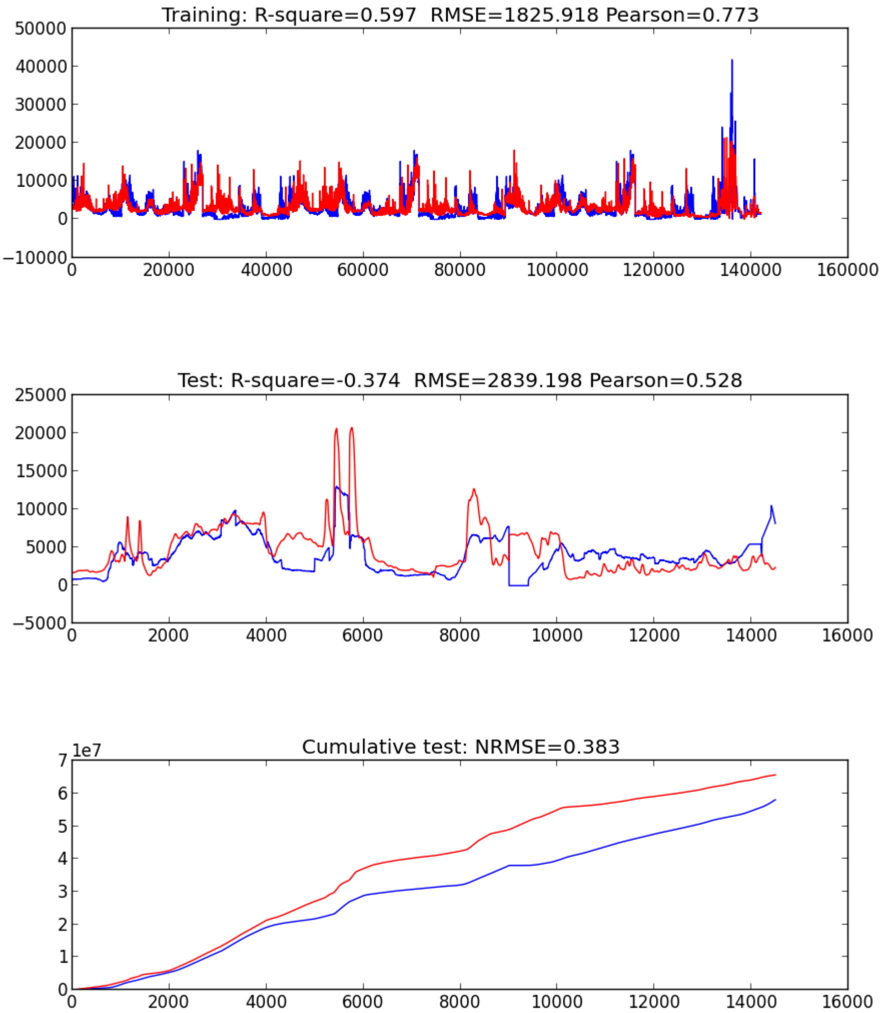

Figure 4: Model performance in Antwerp. The red line represents the model, while the blue line represents the real data from the reference device.

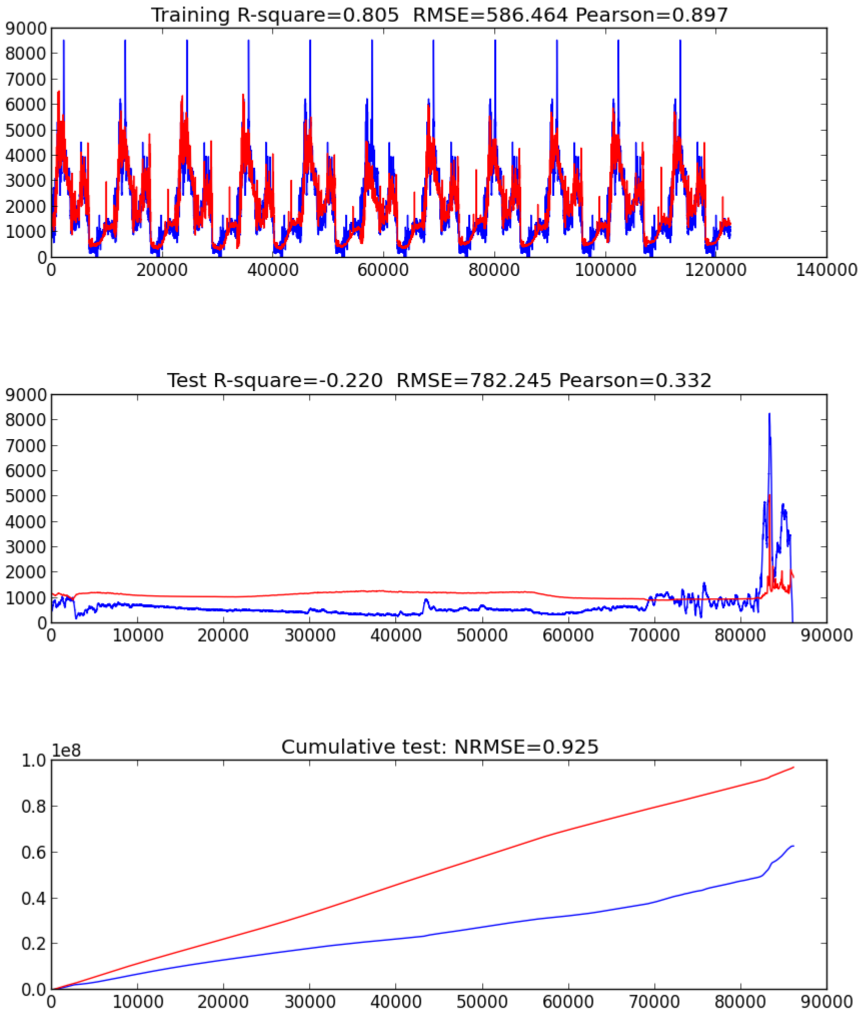

Figure 5: Model performance in Kassel. The red line represents the model, while the blue line represents the real data from the reference device.

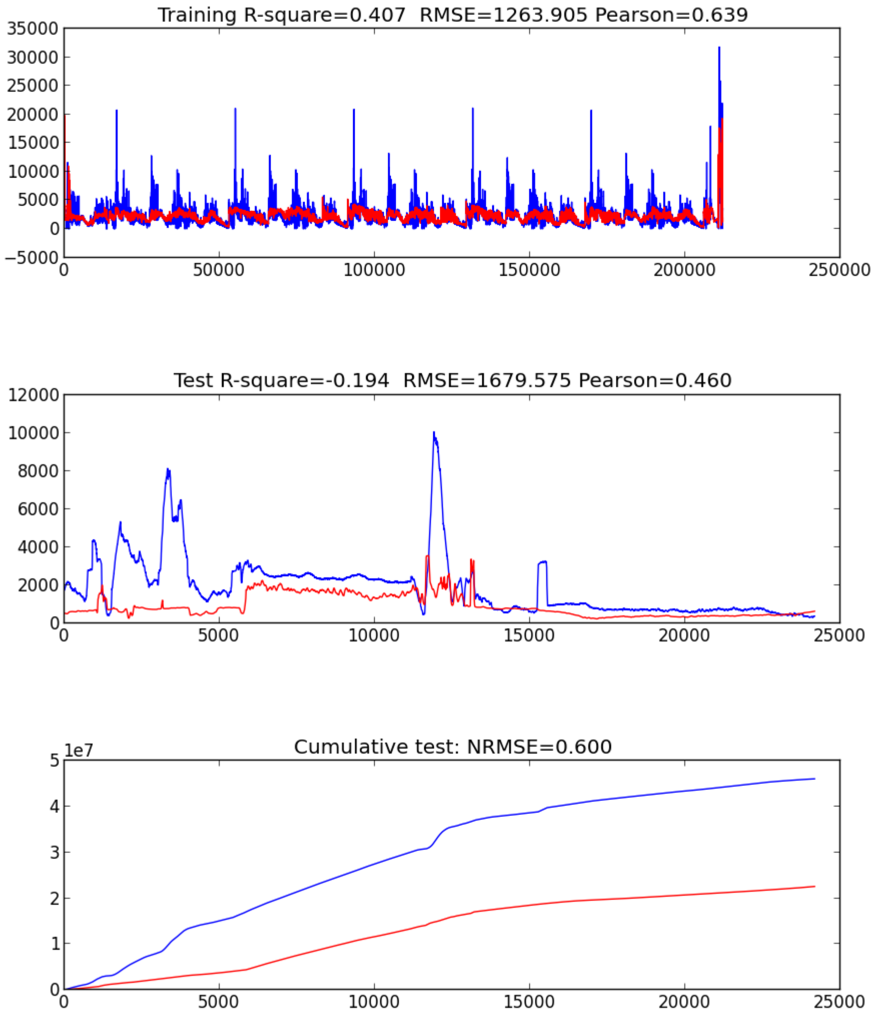

Figure 6: Model performance in London. The red line represents the model, while the blue line represents the real data from the reference device.

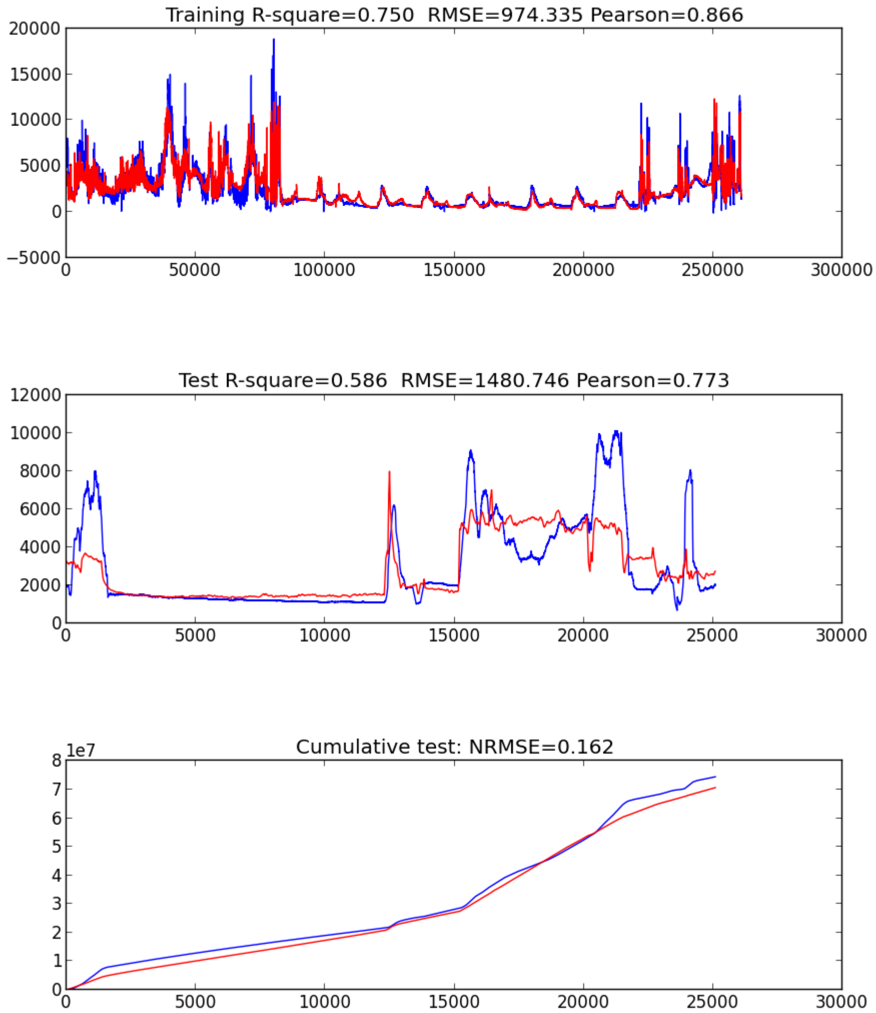

Figure 7: Model performance in Turin. The red line represents the model, while the blue line represents the real data from the reference device.

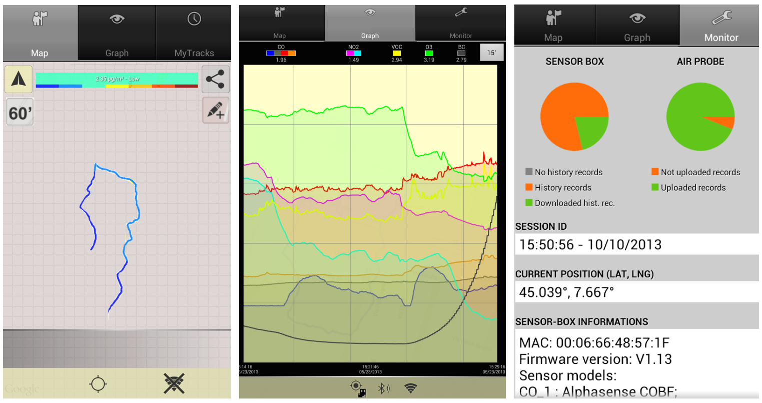

Figure 8: AirProbe screenshots: Live mode. The map view (left) is a representation of the original image, where map data has been removed due to copyright issues.

Graph , where the user can see black carbon evolution and the raw data from pollutant sensors, in a variable time interval ranging from 1 to 30 minutes. The user can query the value registered by each sensor by tapping on the series. The graph is updated every two seconds.

Monitor , where users can access statistics about collected data, connection information, the status of the sensor box and the installed sensors.

3.2 Synchronization mode

In this working mode, AirProbe downloads data from the sensor box and uploads them to the EveryAware server (Figure 9). The sensor box in this case is used as a pure data logger, allowing the user to send data only in suitable conditions (e.g. where battery lifetime and/or connection billing are not a problem).

3.3 Browsing mode

This working mode does not require an active Bluetooth connection to a sensor box. It is composed by three views, accessible from their corresponding tabs:

Map , where the user can see the black carbon levels around his current position (Figure 9), by pressing the "Get nearby BC levels" button. If a track from "MyTracks" tab is selected, this is displayed on the map. The black carbon levels and selected track can be shown together.

Graph , where the raw pollutant and black carbon evolution, calculated for a selected track, are shown. Only live recorded tracks have black carbon data.

My Track , where the list of tracks available on the mobile device is shown. Older tracks are automatically deleted only once they have been uploaded to the server and a configurable time interval since their creation has passed.

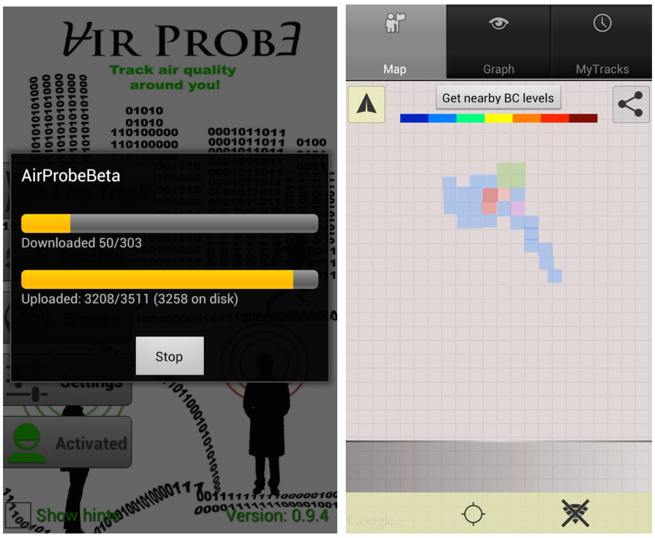

Figure 9: AirProbe screenshots: Synchronization and Black Carbon map. The BC map view (right) is a representation of the original image, where map data has been removed due to copyright issues.

4 The web platform

The personalized air quality data is collected using the AirProbe module of the EveryAware platform which is a social information technology system used by people to communicate and share information. A characteristic of social information technologies is that they often involve very large amounts of data. In fact, the collection, storage, and analysis of different kinds of data within these systems is a crucial point and also an asset, e.g., for companies like Facebook[1]. As a consequence, in order to pave the way towards analyzing and even triggering behavioral shifts within large citizen populations, methods and techniques of acquiring and handling such data efficiently play a central role. The design of web-based infrastructures for this purpose has a great influence both on data quantity and quality, and hence also on the additional value which can be generated by analyzing the resulting datasets. Typical goals during the design process are:

- *Performance:* All infrastructure modules must be carefully tuned for high-performance requirements of processing large amounts of data in a parallel fashion because the involvement of large numbers of users requires responsive interfaces and efficient server backends
- *Management:* The setup and technical realization of experiments and studies among citizens often implies strong efforts on the side of scientists and experimenters. As a consequence, it is desirable to provide reusable and configurable experimentation platforms which can easily be managed.
- *Correctness:* A large-scale collection of data can hardly be expected to provide only correct and consistent results. However, the reduction of noise from the very beginning (i.e., the concrete measurements) is desirable in order to provide a better basis for later analysis.

[1] `http://facebook.com`

Broadly speaking, the relevant data in the context of the EveryAware project can be divided into two classes, namely

1. *objective data*, which stems mainly from sensors and captures information like sound intensity or air quality measurements (as analyzed in this work), and
2. *subjective data*, which comprises context information about the collected data including reactions of humans faced with particular environmental conditions. This comes from annotations that users attach to their measurements and is different from the subjective data we collected through the web game.

The EveryAware platform has been explicitly designed to support subjective impressions in conjunction with sensor data acquisition by introducing an extendable data concept. A central server efficiently collects, analyzes and visualizes data sent from the arbitrary sources. The platform offers a highly flexible way to store and exchange data for Internet of Things applications. A wide variety of meta, location, and content information which can be attached to any data point, a flexible *data processor component* as well as an efficient storage structure are the keys to this task. These mechanisms provide the unique ability to enrich data with contextual information explicitly including subjective impressions. Different collection concepts like sessions to represent time-interval-based entities and feeds to organize data points in a continuous way allow to further introduce semantic relations. This enables the web interface to provide different semantically enriched views on the data, aggregating data globally as well as on a personal level. Allowing users to access this information is a crucial part of the system since it closes the loop from data collection to analysis to pushing information back to individual users and communities which in turn triggers new collection activities. For more information about the EveryAware platforms and its components we refer to Becker et al. [**?**].

4.1 Statistics and visualizations

In the case of AirProbe the visualized information is represented by several views of the data including a map with different information layers as well as several global and personal statistics. The OpenStreetMap-based[2] map view visualizes the collected data on a map which allows for an easy access to the data as well as for obtaining first insights. It provides a quantitative view by aggregating samples using clusters, grids, as well as a heatmap view in order to emphasize the covered area on a global and on a personal level (see Figure 10).

Further statistics calculated by the AirProbe application include summaries like latest overall measurement activity or air quality averages. Also, personal user profiles are available which list measurement sessions giving short summaries regarding those sessions and enabling the user to view and replay them. A personal sessions overview can be seen in Figure 11(a). One view for exploring personal sessions can be seen in Figure 11(b).

4.2 APIC rankings

Additionally, the web interface provided feedback for the users participating in the APIC game by measuring air quality using sensorboxes. The case study was held in order to gather large amounts of air quality samples and behavioral shift patterns using the sensorboxes in the four cities Antwerp, Kassel, London, and Turin.

In order to keep the motivation and competitiveness as high as possible for the teams playing, we implemented a ranking mechanism balancing repetitive sampling and coverage. The map was

[2] `http://openstreetmap.org/`

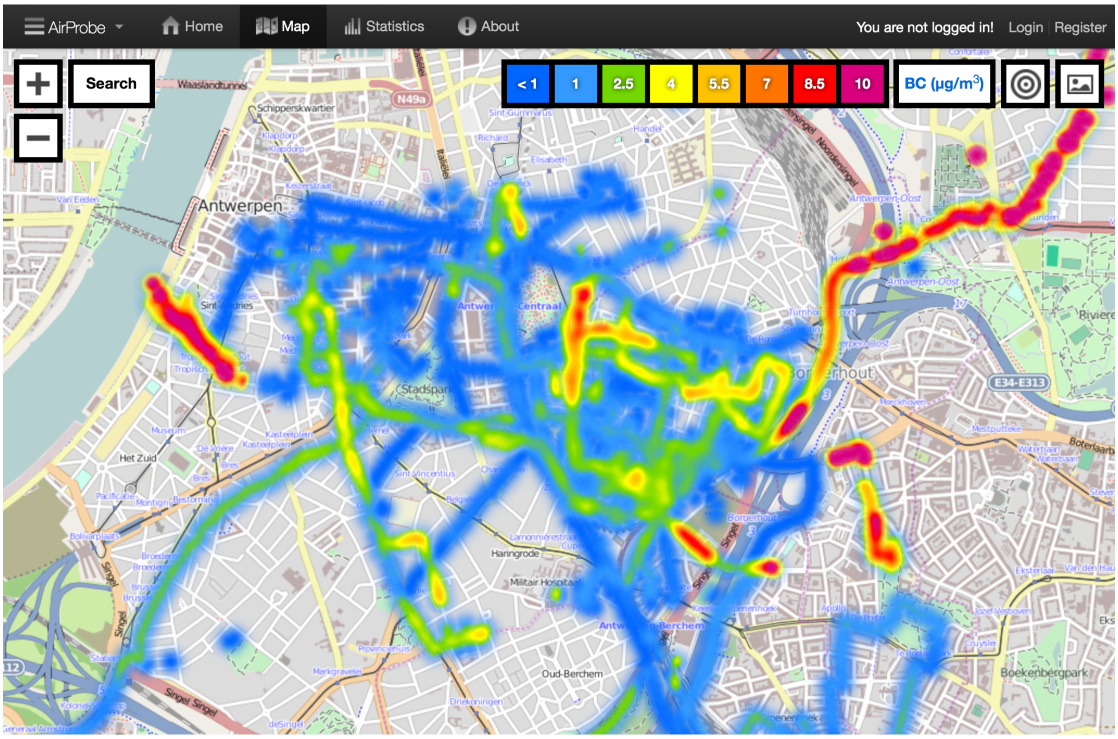

Figure 10: A screenshot of a heatmap on the *map page* of AirProbe. The website map and heatmap were generated using in-house developed tools and OpenStreetMap data (©*OpenStreetMap contributors* for map data, used and redistributed under the CC-BY-SA licence[?]) .

divided into 10 by 10 meter grids. One point was given to a team when sampling within one such grid cell. When a team received a point in a particular cell, the player did not receive a point from this grid cell for half an hour. The results for each city as well as for each team have been visualized and updated in regular intervals on the AirProbe website as can be seen in Figure 12. Figure 12(a) shows the ranking of each city visualizing the coverage and providing several statistics. Figure 12(b) shows a detailed view of the point-coverage of the city.

5 The APIC web game

In order to gather subjective opinions about air pollution in the four cities we decided to follow the *game with a purpose* [?] approach and accomplish the task using a web game. We started designing the game taking inspiration from the specific kind of data we wanted. Our aim was not only to get a map of perceived air pollution but also to study how the perception is affected by objective data. Specifically, we needed to monitor volunteer opinion before, during and after exposure to objective air quality data, obtained by the sensing device. This meant keeping the players engaged in the game for the longest time possible, in order to monitor the opinion shift of each player. Beside this, opinions about air pollution had to be geo-localised so the game had to take place on the maps of the four cities. In particular, for each city we defined a mapping area of approximately 3 km^2, around busy city centre locations.

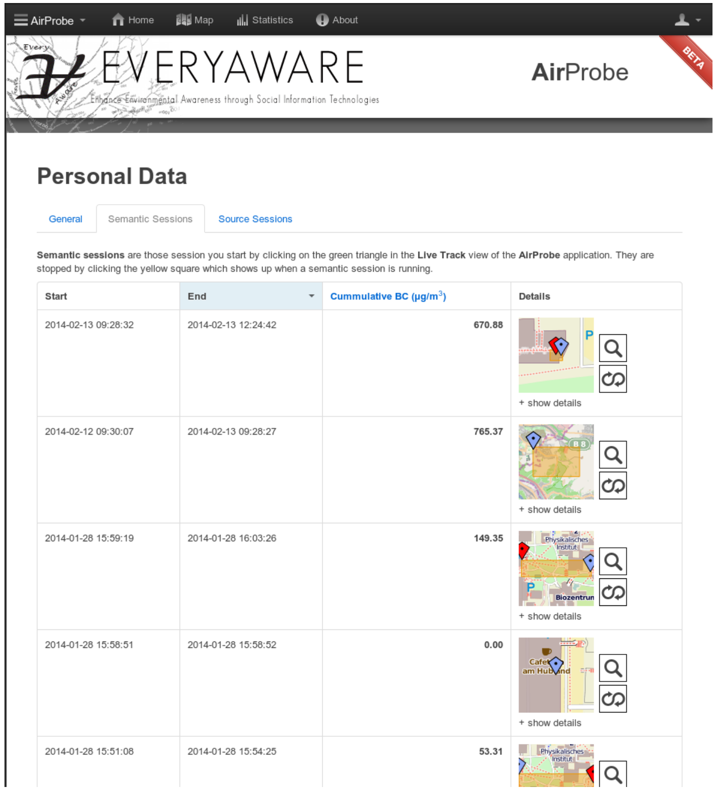

(a) This AirProbe view shows a user's personal sessions.

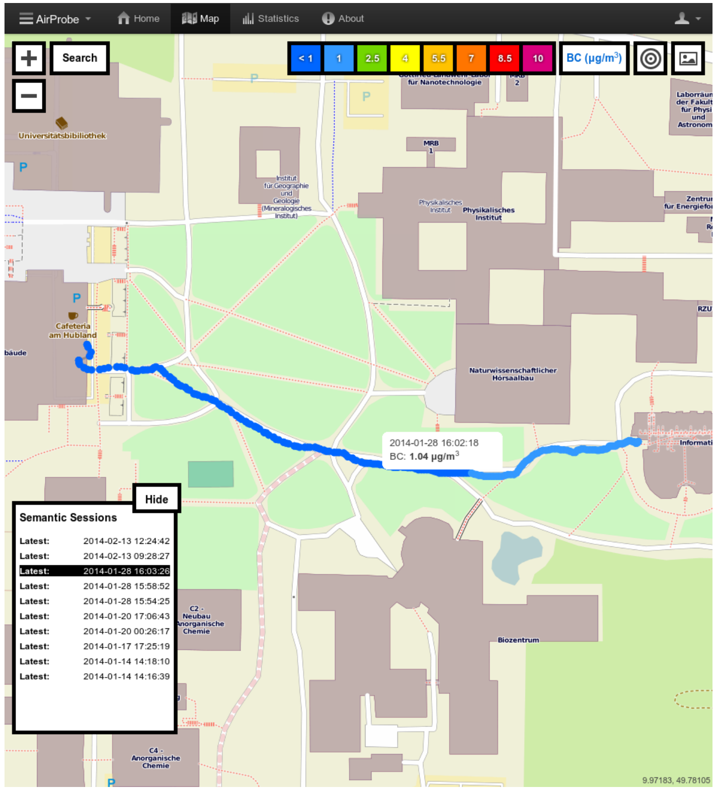

(b) This AirProbe view shows a view for exploring individual user sessions.

Figure 11: AirProbe personal measurement sessions visualizations. The website map and track visualisation were generated using in-house developed tools and OpenStreetMap data (©*OpenStreetMap contributors* for map data, used and redistributed under the CC-BY-SA licence[?]) .

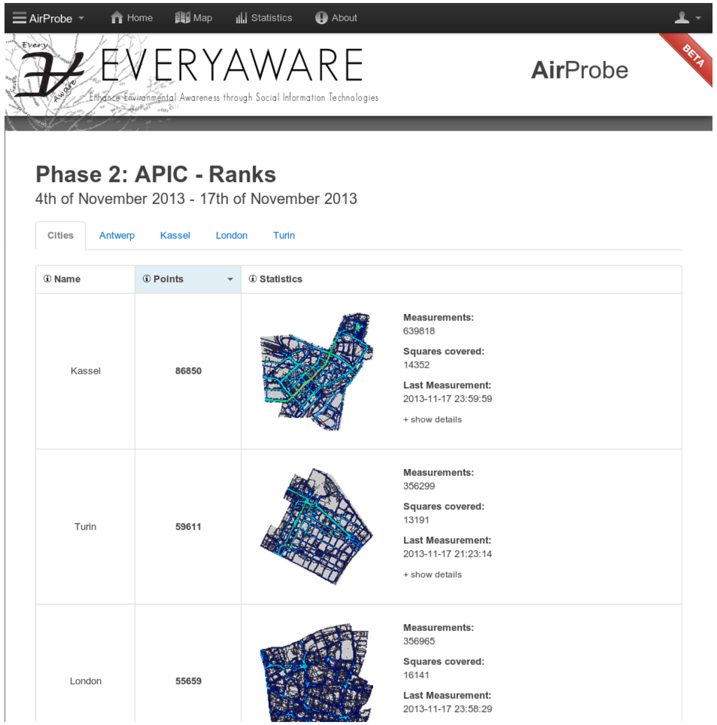

(a) APIC city ranking.

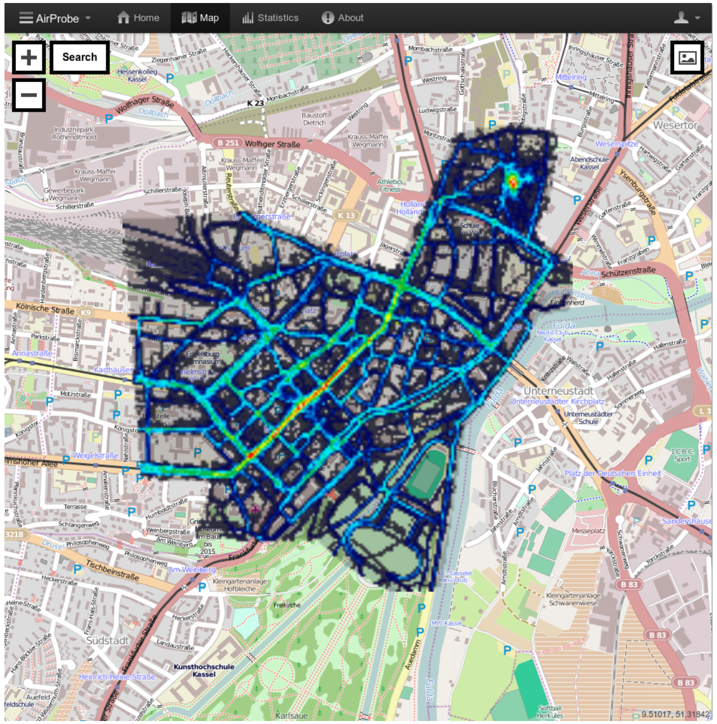

(b) APIC point coverage for Kassel.

Figure 12: APIC ranking visualizations. The website map and heatmaps were generated using in-house developed tools and OpenStreetMap data (©*OpenStreetMap contributors* for map data, used and redistributed under the CC-BY-SA licence[?]) .

Considering all this, the most suitable type of game appeared to be a management simulation, like the famous FarmVille or Harvest Moon. In this type of game the user has the task of managing a given territory. By improving their management performances, the users increase their income in the game. Thus they may access a wider set of features, for example they can expand their territory or buy more objects, all in order to further improve their income. The periodic rhythm of this cycle is marked (in FarmVille-like games) by the time the income is claimable by the player: in order to generate a revenue, an action is required at a given time, spanning from a few seconds to several hours. This mechanism is an incentive to return to the game, in order to gather the results of one's effort.

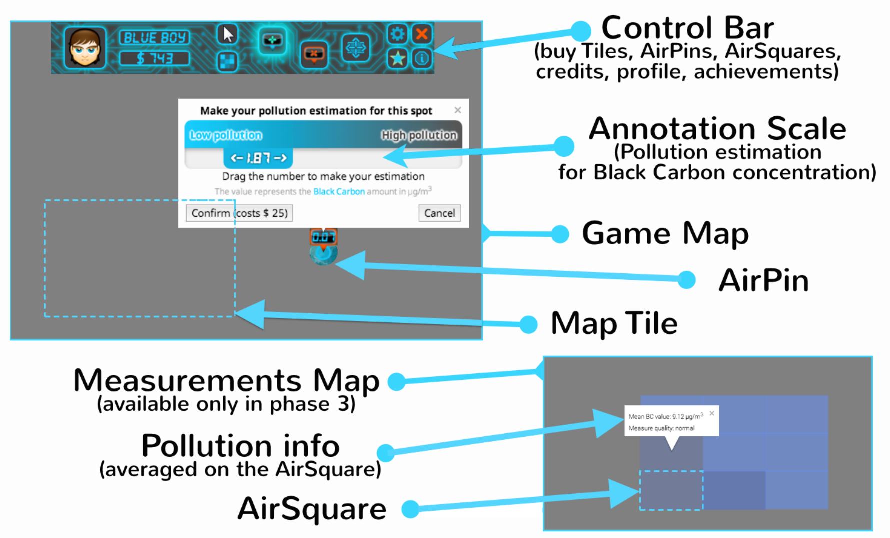

Figure 13: Elements of the game interface, with indication of the main entities and tools. The figure is a representation of the original interface with map images removed due to copyright issues.

The AirProbe web game is a simplified map management game. In Figure 13 the interface of the game is depicted. Players are called to fulfil their role of Air Guardians by annotating the map with AirPins: geo-localised flags tagged with an estimated or perceived pollution level (Black Carbon concentration in $\mu g/m^3$, on a scale from 0 to 10). The game area of each city is divided into tiles.

At the beginning of the game, users are asked to create a profile (by choosing an avatar and a name) and to choose a city and a team. Teams were linked to Air Ambassadors, and were an important part of the competition. Then the volunteer starts from a given Tile of the map of the chosen city. The user can interact by placing (or editing or removing) AirPins or by expanding their territory by buying more Tiles. Each day the AirPins placed generate a revenue based on the precision of the annotation (more details in the following). In order to collect the revenue generated every day by each AirPin, the user has to access the game daily, otherwise the revenue will be wasted. The revenue collected will be added to the user balance, and can be used to buy more AirPins and more Tiles. In order to improve motivation and fidelity, there is a bonus for days-in-a-row accesses and a large set of other achievements. These achievements consist of prizes at given milestones in the game story: a certain number of AirPins or Tiles, precision in the annotation, and so on.

In phase 3 of the case study we made available information about objective measurements gathered with the sensor box during phase 2. We avoided to give punctual information about measurements, otherwise it was likely that users would simply copy the values. So we decided to release aggregated information by introducing a new map partition named AirSquares. Each Tile contains 12 AirSquares, that can be purchased just like AirPins or Tiles. Once users bought an AirSquare, they can see the average pollution value in that area, so the task changes into estimating fluctuations.

5.1 Revenue and feedback

Our case study was divided in three phases. Beside the AirSquare introduction in phase 3, the only change between phases was in the revenue calculation algorithm. We generically said that revenue was related to precision of the annotation. Let us now define the meaning of 'precision' in our context. In phase 1 there were no objective data for comparison, thus we adopted the strategy of matching the AirPins with the estimations of other users within a certain range (30 meters).

The general algorithm of revenue calculation for a certain AirPin f_0 with coordinates (lon_0, lat_0) and value bc_0 was chosen in order to fulfill these conditions:

No data Even if we solved the problem of the lack of data by comparing a user annotation with other user annotations, at the beginning of phase 1 those were missing as well. So, in case of absence of other AirPins within the range, the only choice was to trust the user and give him an average revenue for the AirPin.

Distance In case other AirPins do exist within the range, their distance from the location of f_0 had to be taken into account.

Reliability of the match If an AirPin value matched those of a large number of other AirPins, the revenue had to be large. So the maximum possible revenue is determined by the number of AirPins within the range.

We decided that the most simple and reasonable choice to give revenue for an AirPin f_0 was based on a comparison of the Black Carbon value bc_0 associated with f_0 and the average of all the AirPins F (including f_0 itself) within range of f_0 weighted by their distance to f_0 and rescaled depending on the number of AirPins in range. So, let $F = \{f_0\} \cup \{f_1, \ldots, f_n\}$ be the set of all AirPins within 30 meters from f_0, including f_0 itself and consider the tuples $(bc_i, dist_0(f_i))$ of Black Carbon estimates bc_i and distances $dist_0(f_i)$ from f_0 for all AirPins in range $f_i \in F$. Let bc_F be the weighted mean of all values bc_i in F using a weight w_i defined as

$$w_i = 1 - \frac{dist_0(f_i)^2}{30^2} \tag{1}$$

Let W be the sum of all w_i. Now, we computed the maximum revenue for an AirPin f_0 based on this sum of weights W. We use an inverse exponential function to adjust the maximum revenue (r_{max}) from 30 (when $W = 1$) to about 65 (when $W = 10$) to 75 (when $W > 20$):

$$r_{max} = 30 + 45(1 - 2^{-\frac{W-1}{3}}) \tag{2}$$

We now define the 'error' $e_0 = |bc_0 - bc_F|$ of the estimation for the AirPin f_0 as the absolute value of the difference between the AirPin value bc_0 and the weighted average bc_F of F. Finally, we defined a critical threshold t for the error. If $e_0 > t$ then the revenue will be 0, otherwise the

revenue is calculated using a formula taking the maximum revenue r_{max}, the error e_0 as well as the defnied critical threshold t into account:

$$r_0 = \begin{cases} 0, & \text{if } e_0 > t \\ r_{max}\frac{1-e_0}{t}, & \text{else} \end{cases} \tag{3}$$

The users only had an aggregated view on their revenue, i.e., only the cumulative value for the whole ensemble of their AirPins was shown. The only feedback regarding single flags was a red sign for flags that were not generating any revenue.

As we said, the revenue algorithm has been different in each of the three phases:

Phase 1 The threshold for the error was very tolerant (5 μg/m^3) in order to make the game easy at the beginning.

Phase 2 The threshold was made smaller (2.5 μg/m^3), in order to make the game more challenging and keep users engaged.

Phase 3 The threshold was unchanged but real measurements from sensor boxes were used instead of other players annotation to calculate the revenues.

Users were not informed about the details of the algorithm. They were just asked to try to be precise. Every day ranks were published. In order to boost motivation, we introduced a set of prizes to be given at the end of each phase and in each city. We considered two main metrics for the ranks: the total revenue of the last day of play and the number of days played in each phase (fidelity).

6 Recruiting activities

In order to recruit participants for the study each city adopted their own recruitment strategy alongside publicity via the APIC Facebook page, Twitter and the project website which was used across all the cities. University mailing lists were used to recruit volunteers in each location, excluding Antwerp, who alongside Kassel were the only cities to use external email mailhosts. In Antwerp, where similar air quality monitoring activities have previously been carried out, the challenge was advertised via a specific mailing list which included volunteers from earlier monitoring campaigns, traffic organisations, environmental agencies and interest groups, and communities working on sustainability issues. The advertisement included a link to a participation form that included several questions which were used to gain some ideas on the degree of interest of the participants in air quality monitoring and on the potential temporal coverage expected from monitoring activities. Kassel was the only city to release newspaper articles as part of their recruitment strategy; Turin and London gave talks during classes and a varying number of posters were distributed within university campuses in Kassel, Turin and London.

Interested individuals were asked to contact the relevant project team members and following the initial call for participation meetings were scheduled, specifically for Air Ambassadors, to explain the study in more detail and to provide guidance on using the sensor box. A summary of the number of Air Ambassadors recruited for each location is detailed in Table 6 below. The results show that using existing mailing lists, whether within a university or across other networks, was the most successful approach to securing volunteers in three of the four cities. In Turin, however, public talks proved to be the most successful.

	Antwerp		Kassel		London		Turin	
Method	Responses received	Final volunteers	Responses received	Final volunteers	Responses received	Final volunteers	Responses received	Final volunteers
Mailing list	32	19	7	5	48	30	8	2
News paper	-	-	3	2	-	-	-	-
Talks	-	-	2	1	1	1	11	8
Posters	-	-	-	-	-	-	-	-
Other	-	-	-	-	-	4	-	-

Table 6: APIC recruitment methods and resulting volunteer participation across the four cities.

6.1 Incentives

One of the aims of the AirProbe International Challenge (APIC) was to investigate participation patterns of volunteers for environmental monitoring studies via a web-based *game with a purpose* [?] and competition approach, combining online and offline activities. In addition, comparison of the various incentives offered across the four Case Study areas (London, Antwerp, Kassel and Turin) was explored.

The incentives offered to Air Guardians in each city were as follows: the player with the highest revenue at the end of each phase received a backpack; those ranked second to fourth t-shirts and the most active players also received a backpack. The winning metrics, as outlined in the earlier section, were calculated based on the revenue generated by the AirPins in the last day of play of each phase and fidelity based on the largest number of consecutive days played. Deviations from these incentives were made in Turin where five t-shirts and one backpack were offered for the highest revenue and three t-shirts and one backpack for fidelity. In Kassel an additional incentive was offered to Air Guardians based on the best precision (3 x €50 Amazon vouchers) and the largest most active team with at least three active members playing over a minimum of 21 days (3 x €50 Amazon vouchers). In Antwerp there were no specific incentive schemes and the only place in which prizes were mentioned was on the webpage which stated prizes were on offer for participants taking measurements and for the most active and best gamers.

In each of the four cities all Air Ambassadors were given solar panel backpacks for their contributions and variations across the four cities were as follows:

In London all were given T-shirts and shared £100 Amazon vouchers between each team (a total of 10 teams varying in size from two to six participants). The team who obtained the best temporal/spatial coverage won a sensor box in phase 2 and the winning team overall, defined as having the best temporal/spatial coverage and the largest number of active Air Guardians, received £400 in Amazon vouchers.

Kassel adopted a stricter criterion which in phase 2 offered twelve lots of €50 Amazon vouchers for those who carried out one hour of monitoring for at least seven days; twelve lots of €20 vouchers for those who carried out one hour of monitoring for at least seven days and who fell within the top 50% best ranked Ambassadors world-wide and €250, or a sensor box, for the best temporal/spatial coverage and at least 1.5 hours of monitoring completed for nine days. In phase 3 €250 Amazon vouchers were offered to the Ambassador with the best temporal/spatial

coverage and 1.5 hours of monitoring for nine days; €50 for second place and €20 for third with the best temporal/spatial coverage and at least one hour of monitoring for seven days

Turin offered T-shirts to all Ambassadors and two sensor boxes (one for phase 2 and one for phase 3) to those with highest coverage. In addition they gave final prizes (3 Amazon vouchers €75, €50 and €25) to Ambassadors with the best performing teams, using a combined criterion for evaluation (number of measurements, coverage and game activity for the Ambassador's team). These final prizes were however not advertised to the participants before the end of the challenge.

Antwerp did not specify any specific reward scheme.

The overall challenge winner across all cities was offered EveryAware T-shirts for their effort.

The influence of the different strategies regarding incentives are somewhat visible when analysing the data in the next section.

7 Data analysis

	Total geo-localised	Additional without location	Antwerp	Kassel	London	Turin
Number of measurements	6,615,407	3,326,956	318,537	2,929,345	1,115,828	1,592,912
Number of tags	742	16	3	32	606	11

Table 7: Number of measurements and tags during the test case. Details for each of the four locations.

The analysis presented in this work is based on a large amount of air quality measurements collected using the EveryAware sensorbox during four weeks (phases 2 and 3 of the AirProbe International Challenge) in four European cities. Table 7 summarises the number of data points and tags collected. This shows that in Europe there were over 6 million measurements performed, with Kassel displaying the largest activity. This could be due to the fact that volunteers in Kassel were offered significant monetary rewards for their activity, unlike the other locations. The number of annotations is largest in London, which is due to the instructions they received which underlined the need for subjective annotations.

For further insight into the range of measurements obtained, we show daily (Figure 14) and hourly (Figure 15) numbers of measurements at each location. The different cities show different behaviour. In Antwerp and London, the activity decreased significantly during phase 3 of the challenge. This shows that users were mostly interested in mapping the main area of interest in the challenge. In Turin, however, activity increased during phase 3, which may indicate that volunteers were particularly interested in a different area than that chosen for phase 2, and in monitoring their own daily exposure. In Kassel, activity is more or less stable, with a slight decrease in the last week.

Daily patterns (activity per hour) show afternoon peaks for each location. For Kassel and Turin there is a significant amount of data collected during the night, showing an increased interest again in monitoring and collecting a large number of points.

Besides the general number of measurements coverage patterns are also important. The main text discusses overall coverage, both in time and space. Here we provide more details for

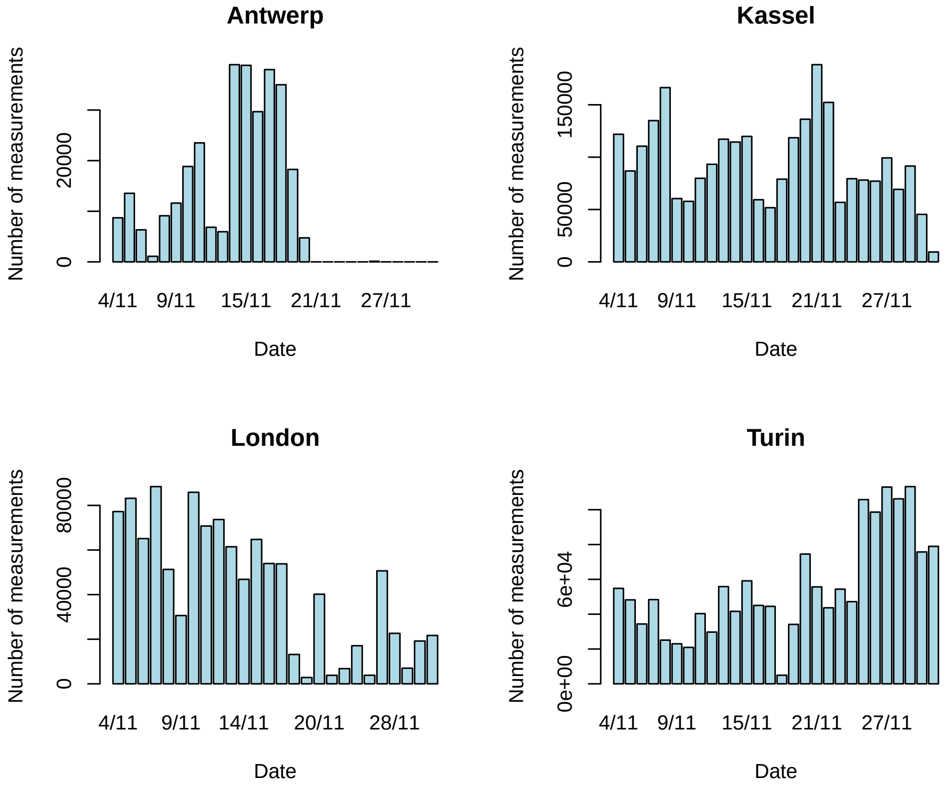

Figure 14: Number of data points per day in the four locations of the challenge.

the different locations, in Table 8, in an aggregated manner: for each location we show the total number of 10 m ×10 m squares covered (space coverage) and the average number of measurements (rough measure of time coverage). The table shows that coverage follows the same trend as the number of measurements (Table 7): the highest coverage is achieved by Kassel, which also won the challenge, and lowest by Antwerp. This applies both to the space dimension (surface covered at least once) and time (number of repeated measurements in an area). In total, volunteers covered over 24 km^2, and each 10 m ×10 m tile contained on average 24 measurements.

	Europe	Antwerp	Kassel	London	Turin
Surface covered in m^2	24,330,700	1,906,500	8,373,400	6,996,000	7,054,800
Average number of measurements per $100m^2$	24.14	17.01	34.71	15.36	22.23

Table 8: Coverage obtained in Europe and at each of the four locations.

For an improved qualitative image of the type of coverage patterns in the different weeks of the

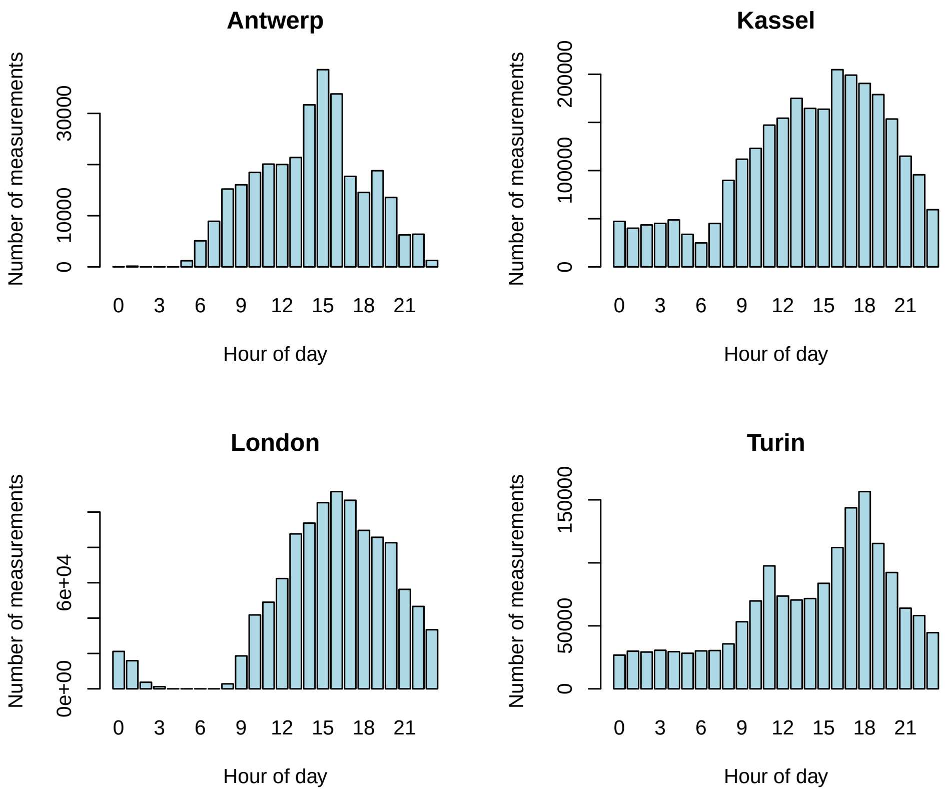

Figure 15: Number of data points per hour of the day at the four locations of the challenge.

challenge, we provide two examples from two different teams in Turin (Figures 16 and 17). These validate the observations made in the main text: during the first two weeks of measurements teams explore more, in their aim to cover the area of interest, i.e., the predefined mapping area, as well as possible. In the last phase, however, when no mapping area exists, they perform repeated measurements on their daily tracks, with reduced space exploration. This pattern is important for further analyses, since the space/time coverage appears to be much better when the area is restricted.

For a better view of the evolution of measured pollution levels between phases 2 and 3, Figure 18 shows the distribution of BC for the different locations, compared in the different phases. We use notched boxplots, which show minimum, maximum and quartile values for the data: the box represents the range of the data between the first and third quartile. The notches show confidence intervals (if these do not overlap, differences between the distributions are significant). In the plots presented here, the notches are so small that they are invisible. The plots also contain information about the size of the different datasets: the width of the boxes is proportional to the square root of the number of data points represented. In Kassel, volunteers were grouped into two groups in phase 3: the first group (g1 - three sensor boxes) had as a task to avoid highly polluted areas, while group g2 (6 sensor boxes) had no task other than using the sensor box where they wished. This in order to test whether any learning appears during measurements.

For Antwerp, volunteers collected much higher BC levels in phase 3. In London, although

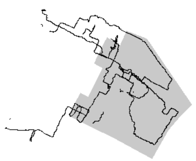

(a) Week 1.

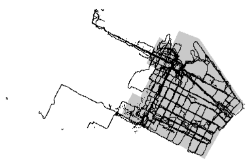

(b) Week 2.

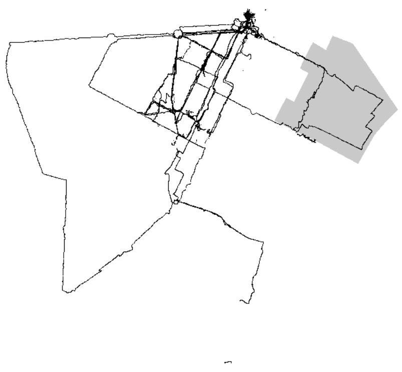

(c) Week 3.

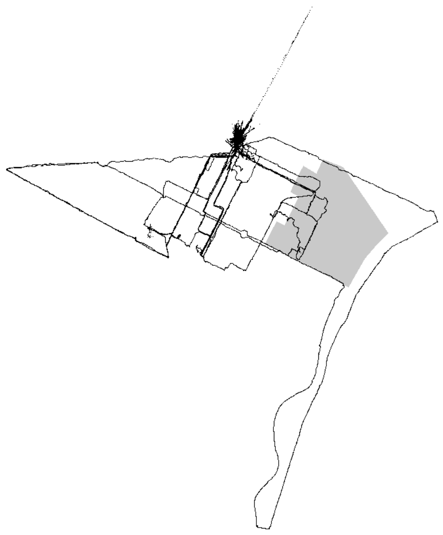

(d) Week 4.

Figure 16: **Coverage for group "ggwp"** over the four measuring weeks of the APIC challenge. The grey area indicates the predefined mapping area.

means are not larger, the maximum levels achieved are larger in phase 3. However, for these two cities data in phase 3 is rather limited compared to the other locations and to phase 2 (as shown by the width of the boxplots in Figure 18 and in Figure 14). For Turin, an increase in the measured pollution levels is clear again. So, for all three locations, there is a good indication that

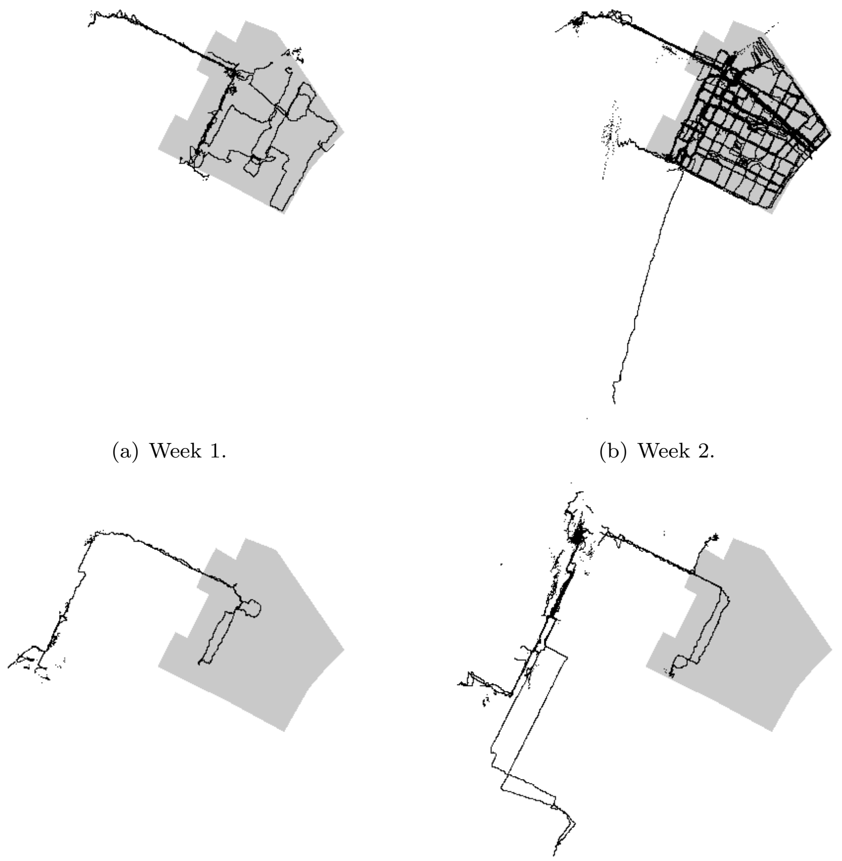

(a) Week 1. (b) Week 2.

(c) Week 3. (d) Week 4.

Figure 17: **Coverage for group "TUX"** over the four measuring weeks of the APIC challenge.

volunteers concentrated more on high pollution levels in the 3rd phase of the challenge: when they were allowed to explore, the aim was to identify highly polluted locations.

For Kassel, the group tasked with minimising their exposure (g1) displays on average larger BC levels compared to the other group. Maximum values appear, however, to be lower compared both to the previous phase and to g2. This may indicate that volunteers have only learned how to avoid extreme pollution levels, but still cannot discriminate when it comes to average behaviour.

Of course, pollution levels themselves may change from one day or period to another, making evaluation of user behaviour difficult. For instance, if a user appears to measure higher values in time, this could be either because of a shift in his personal interests, or because pollution itself increases. For the period of the challenge, PM10 data (particulate matter with an aerodynamic diameter under 10 μm) were available from official monitoring stations for all locations, while BC only for Antwerp [**?**, **?**, **?**, **?**]. Although BC is mainly represented in the small diameter ranges

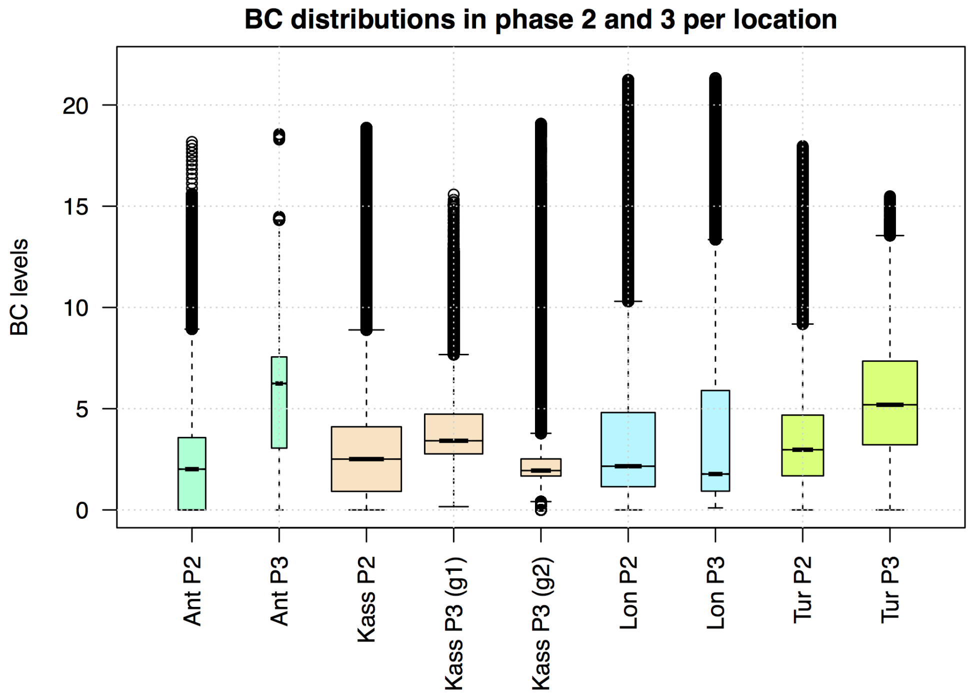

Figure 18: **Pollution levels per location compared in the two phases.** The distribution of BC levels (in $\mu g/m^3$) are shown for the two measuring phases of the challenge, phases 2 and 3, separate for each location. For phase 3 Kassel's AirAmbassadors are split into two groups with different objectives. Group 1 (g1) was supposed to avoid strongly poluted areas. Group 2 (g2) had no specific goal.

of PM, PM10 data were used for comparison with the measurements, due to higher availability, since they give a good indiction of the general level of pollution. In Figure 19, we compare the daily average PM10 with average BC values obtained by our volunteers. In Antwerp, we also show official average BC levels. As the figure shows, BC levels measured by our volunteers are within a good range compared to PM10 values. Differences are comparable to those observed between reference BC and PM10 in Antwerp and may indicate some particular interest of the volunteers. Also, no increase between phases 2 and 3 is visible in PM10 data. Table 9 shows average PM10 for all locations for phases 2 and 3 (for Antwerp we also show BC). This confirms that no significant increase in overall pollution levels appeared from phase 2 to phase 3.

Table 9: Average official pollution levels for the four locations in phase 2 and phase 3 of the challenge.

	Antwerp PM10	Antwerp BC	Kassel PM10	London PM10	Turin PM10
Phase 2	29.25	4.33	26.2	26.73	50.42
Phase 3	28.29	3.03	28.1	26.05	39.66

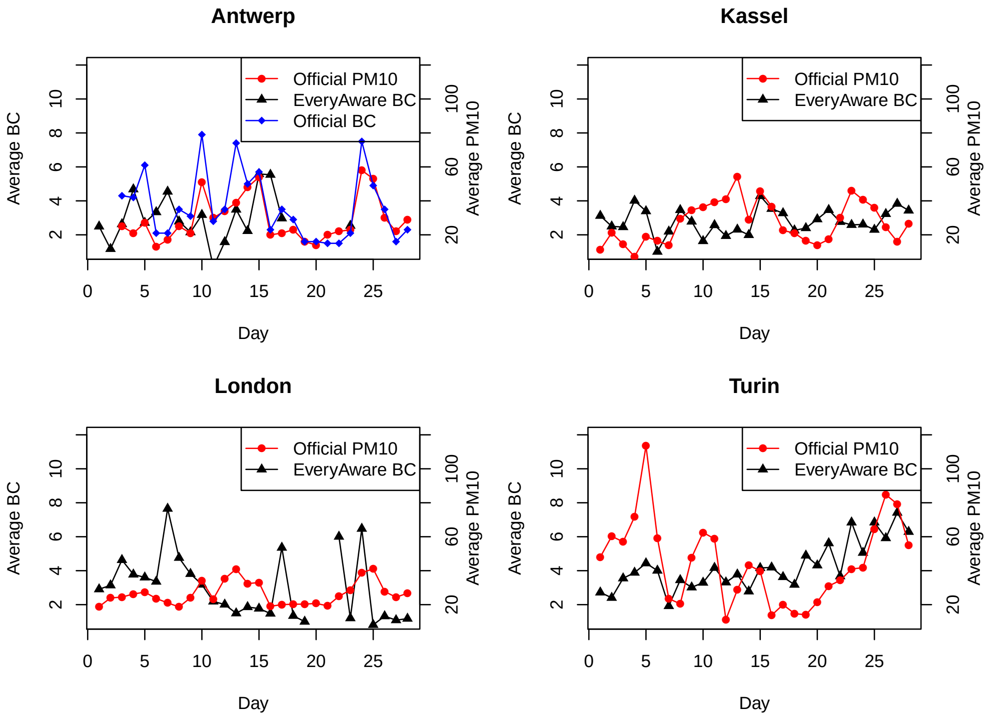

Figure 19: Comparison of measured average BC to PM10 and BC reference measurements in the areas of interest.

7.1 Opinion evolution model

This section revisit the APD graphs from the main text aggregated according to each phase reported in Figure 20.

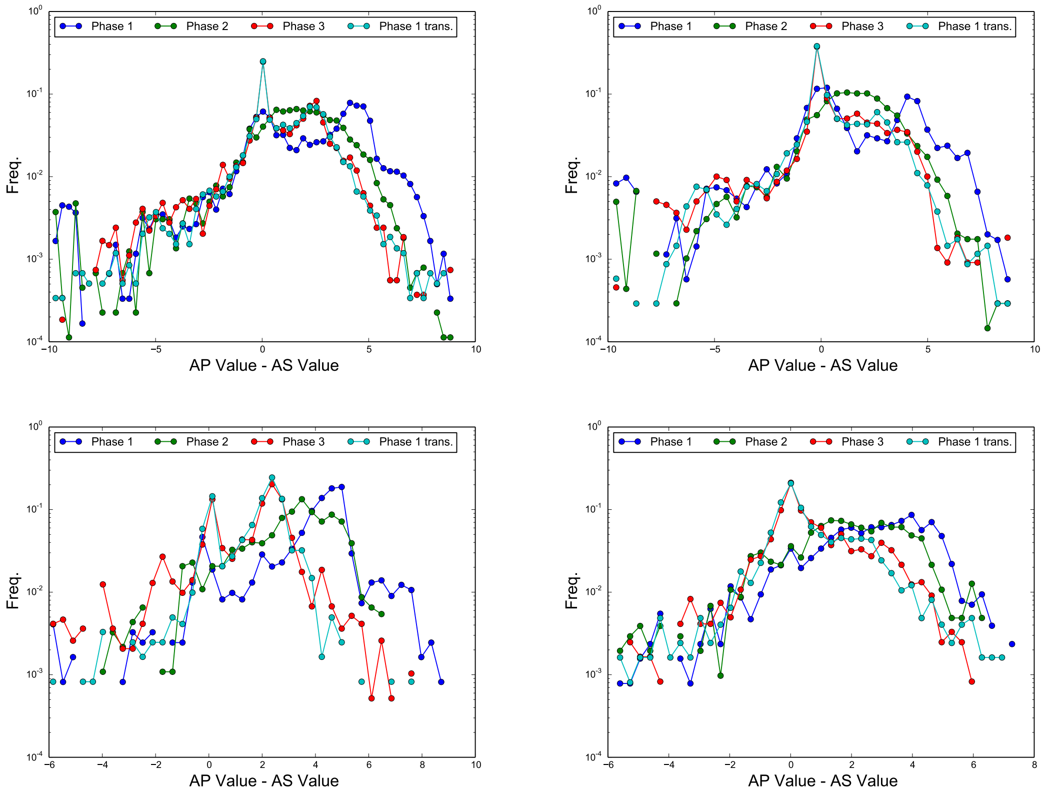

Figure 20: Clockwise, from the top left: the APD histogram for the overall, for Kassel, for Turin and for London in each phase of the challenge and with an estimation of phase 3 data obtained from phase 1 data through the transformation defined in Eq. (5).

If we look at phase 3 histograms two main features attract our attention: a narrow peak at 0 and a strongly asymmetric structure. The first feature was somehow expected since players are trusting the AS (AirSquare) values shown in the AS, and they are annotating accordingly. Fortunately, the peak at zero is not delta like, which would be expected if users were copying the AS value. Rather, players still have their opinion on the environment and keep it despite the on field measurements. This may happen because they are really trying to follow the basic ideas of the game, but also because copying it is not the best strategy, since the AS value is aggregated, i.e. it is the average of all sensor box measurements taken in the corresponding AS, while the real measurements used for revenue calculation were punctual values which could be substantially different. So the shape of the distribution around zero seems to be caused by users learning the most likely air quality value and trying to estimate fluctuations. But graphs in Figure 20 show something more. There is a clear asymmetry for phase 3 distributions, since the great part of APD values fall in the positive range. This could be a consequence of the fact that AS values were around 3 μg/m^3 so there was a 30% probability to underestimate that value and 70% to over estimate, but if we look at the phase 1 distributions, this asymmetry effect seems better explained by a sort of memory effect or inertia of players in changing their opinions. This hypothesis seems realistic if we look at the London graph. The main peak around 4 μg/m^3 is still present in phase 3, although it is shifted. In order to measure this effect we defined a transformation that takes into the account both features just discussed: the accumulation around 0 and the shift. Let us consider a given set of opinions o_i about a certain number of topics provided by a certain number of subjects. At a given time those subjects are exposed to values h_i, which are perceived as hints of the true values. We are interested in what happens to the difference between opinions and hints before and after the exposition, to understand how this information will affect the opinion structure. To this aim, we define the set of differences d_i between the opinions and the relative hints and analyse the distribution of those differences before and after the exposition. Obviously, the variation of the differences is only due to the variation of the opinions. As we said, we want to reproduce the phenomenon of the accumulation around the hints (i.e., $d_{aft} \sim 0$) and the shift of the general opinion, that we will try to describe as a sort of rescaling (i.e., $d_{aft} \sim d_{bef}/r$ where r will be the rescaling constant). Which of the two phenomena will take place will be decided randomly: with a given probability p_0 the opinion will reset around 0, otherwise, with probability $1 - p_0$, the opinion will just be rescaled. Finally, around this two attractors we add a certain amount of noise. We decided for a Cauchy distribution $C(X)$ centered at 0 in one case and at d_{bef}/r in the other, i.e.

$$C(x; \mu, \gamma) = \frac{1}{\pi\gamma\left(1 + \left(\frac{x-\mu}{\gamma}\right)^2\right)} \tag{4}$$

where μ is the average (and the center of this symmetric distribution) and γ represents a scale factor. It is worth to note that the variance of this distribution is not defined, since the second momentum of the distribution does not converge. This choice seems reasonable because tails seem to be power law-like rather than gaussian-like, as the log plots in Figure 20 show. Let us define our transformation and its effect on the difference d_{bef} between the opinion and the hint before the exposure. According to the rules we stated earlier, d_{aft} will be distributed according to this density function:

$$T(d_{aft}; d_{bef}, p_0, r, \gamma_0, \gamma_r) = \begin{cases} C(d_{aft}; 0, \gamma_0) & \text{with prob. } p_0 \\ C(d_{aft}; d_{bef}/r, \gamma_r) & \text{with prob. } 1 - p_0 \end{cases} \tag{5}$$

The transformation we just defined introduces four parameters:

- p_0, which is the probability that the old opinion is reset around $d = 0$; thus, with probability $1 - p_0$, the opinion shows a certain inertia; this resistance to change causes a shift toward the hint instead of a complete reset;
- r, the rescale factor quantifying the shift of resilient opinions;
- γ_0 and γ_r , the γ scale factors for the Cauchy distributions centered respectively at 0 and at d_{bef}/r introduced to add a realistic noise.

We used our data to infer the parameters of our model for Kassel, London, Turin and for the complete set of data. If we apply the transformation to phase 1 data, we get an estimate of phase 3 distances between opinions and hints. Then, to evaluate how good is the estimate, we use a two sample Kolmogorov-Smirnov two sided test. This kind of test gives as result the probability p_{val} that the hypothesis that the two samples are drawn from the same distribution cannot be rejected. Usually, a value below 5% means that the hypothesis has to be rejected otherwise the hypothesis is likely to be true. If the p_{val} is around 10% the two samples come from two distribution which are, in any case, very close. Above 30% the samples can be considered with a good degree of confidence as coming from the same distribution. We explored the space of parameters with 10% steps and repeating the test 100 times to find the combinations with the highest p_{val} for Kassel, London, Turin and for the overall. These optimal combinations are reported in Table 10 with the relative results for the Kolmogorov-Smirnov test.

Table 10: Parameter combinations with the highest p_{val} resulting from the Kolmogorov-Smirnov test. Parameter space has been explored with 10% steps and each configuration has been tested 100 times. The average p_{val} is reported. Some peaks in the tails for London compromised the test, causing as a result unsatisfying values for the parameters. We reduced the range in the most meaningful area, which is $(-1:4])$. We found the best parameters testing only this area, obtaining a remarkable result ($p_{val} = 27\%$). Then we made again the test reintroducing neglected data, obtaining a $p_{val} = 9\%$ which is still a satisfactory result.

dataset	p_0	r	γ_r	γ_0	$< p_{val} >$
Kassel	0.336	1.62	0.381	0.0138	0.192
London	0.147	1.90	0.100	0.030	0.267 (0.087)
Turin	0.583	1.56	0.304	0.300	0.417
Overall	0.204	1.767	0.28	0.015	0.262

From Table 10 it appears that the reset of the opinion around the hint does not happen so often. In London, for example, it is almost a secondary effect. In the *best* case, Turin, the reset seems to be there slightly more than in half of the cases. We also reported in Figure 20 an estimate of the APDs for phase 3 obtained by applying the transformation 5 with the *optimal* parameter combination to the data of phase 1. The similarity between the estimate and phase 3 real data is pretty clear.

It is very likely that Eq. (5) is not the real transformation of the opinion due to the subjects' exposure to hints. We made strong assumptions and we reduced our data set to focus on the interesting part. Also, we are analyzing and modeling the phenomenon on a very narrow time scale (weeks) without knowing almost anything about the others (for example, if we considered months the dynamics could be potentially extremely different). Despite these considerations, the results we showed point out with sufficient reliability that the main components are there. The model we referred to helped us to measure how our volunteers were influenced by the hints we gave them. We may now affirm with a certain degree of confidence that even when people do not trust completely the AS values, they still get influenced by them. Another way to see this is that, even if people do not reset their opinions, the space itself in which their opinions are

arranged is deformed by the exposure to hints. Obviously these considerations are justified if the subjects consider the source of the hints as *objective*. In other cases, for example, if volunteers are told that opinions come from other volunteers, completely different dynamics are expected to come into play.
